# Cross-Scale Multimodal Imaging for Organic Matter in Extraterrestrial Samples

**DOI:** 10.1021/acs.analchem.4c05804

**Published:** 2025-03-18

**Authors:** Mingtan Dong, Wei Yang, Jialong Hao, Xiaofei Jia, Ou Yang, Michael K. F. Lo, Bobo Cao, Sen Hu, Yangting Lin

**Affiliations:** † Key Laboratory of Earth and Planetary Physics, Institute of Geology and Geophysics, Chinese Academy of Sciences, Beijing 100029, China; ‡ University of Chinese Academy of Sciences, Beijing 100049, China; § Waters Corporation, Beijing 100076, China; ∥ ULVAC-PHI Instrument Co. Ltd., Nanjing 211102, China; ⊥ Photothermal Spectroscopy Corporation, 325 Chapala Street, Santa Barbara, California 93101, United States; # Department of Chemistry, Tsinghua University, Beijing 100084, China

## Abstract

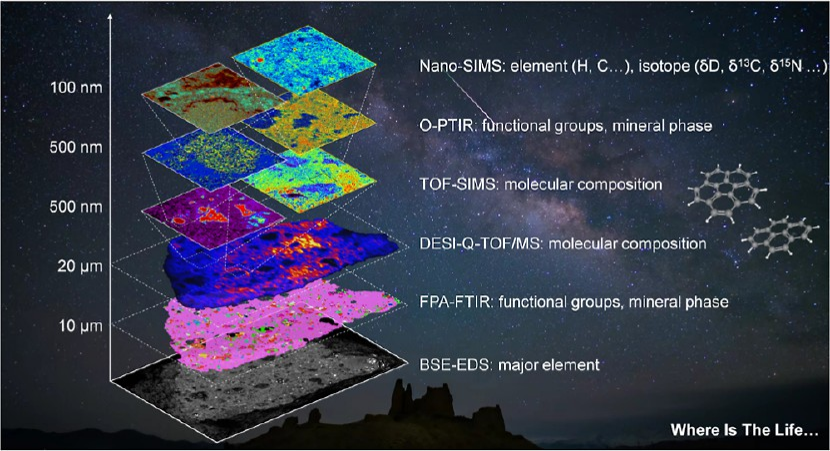

The analysis of extraterrestrial organic matter in samples returned by space missions provides a unique opportunity to study prebiotic chemistry. A comprehensive understanding of the occurrence and composition of organic matter is fundamental to unraveling its origin and evolutionary history. However, the scarcity and complexity of these materials pose considerable analytical challenges. Here, we developed a cross-scale multimodal imaging workflow that integrated mass spectrometry imaging (MSI) and vibrational spectroscopy imaging, including desorption electrospray ionization coupled quadrupole-time-of-flight mass spectrometry (DESI-Q-TOF/MS), time-of-flight secondary ion mass spectrometry (TOF-SIMS), nanoscale secondary ion mass spectrometry (NanoSIMS), focal plane array-Fourier transform infrared spectroscopy (FPA-FTIR), and optical photothermal infrared spectroscopy (O-PTIR). This workflow was applied to the Murchison meteorite, with the objective of establishing spatial associations between mineral phases, molecular composition, functional groups, and isotopic composition on a scale from the millimeter to the submicron. The spatial resolution of DESI has been improved from 100 to 200 to 20 μm, enabling spatial correlation with other imaging techniques. For the first time, the enrichment of organic matter—including CHN, CHO, and CHNO compounds and polycyclic aromatic hydrocarbons (PAHs)—in fine-grained rims (FGRs) surrounding silicate chondrules has been observed. Furthermore, the cross-scale multimodal imaging also reveals differences in organic matter composition between Ca-carbonate and phyllosilicates, as well as spatial heterogeneity within the latter. This workflow provides a new paradigm for studying the complex occurrence and composition of organic matter in various research fields, enhancing our understanding of prebiotic materials in the solar system.

## Introduction

Recent space missions, including Hayabusa, Hayabusa2, and OSIRIS-REx, have successfully returned samples from the Itokawa, Ryugu, and Bennu asteroids.^1^ These returned asteroid samples allow for detailed analysis in Earth-based laboratories and provide an opportunity to investigate the origin, formation, and evolution of prebiotic molecules in the solar system.^1^ To comprehensively study extraterrestrial organic matter, it is necessary to investigate its occurrence (i.e., its relationship with inorganic minerals), elemental composition, molecular formulas and structures, and isotopic information.^2−4^ However, the organic matter in these samples is generally small in size and has complex compositions, posing a challenge for analytical techniques. Therefore, it is imperative to obtain as much information as possible—both global and detailed, organic and inorganic—while minimizing the consumption of these limited samples. It is unlikely that all information can be obtained through a single technique. Integrating multiple techniques to avoid the “blind men and the elephant” dilemma in analyzing complex natural samples is thus an urgent issue.^5^

Previous analyses of organic matter in asteroid Ryugu samples can be divided into two approaches.^2−4,6−8^ First, techniques such as Raman spectroscopy, Fourier transform infrared spectroscopy (FTIR), and atomic force microscope-based infrared spectroscopy (AFM-IR) are employed to investigate the functional group composition in situ. Second, techniques such as Fourier transform-ion cyclotron resonance mass spectrometry (FT-ICR/MS), ultrahigh-performance liquid chromatography with fluorescence detection and high-resolution mass spectrometry (LC-FD/HRMS), and desorption electrospray ionization (DESI) coupled with HRMS are employed to elucidate the diversity of organic molecules, such as polycyclic aromatic hydrocarbons (PAHs), carboxylic acids, and amino acids.^3^ However, these methods often require extraction of the organic matter, resulting in the loss of spatial information.^3^ Although DESI, as a mass spectrometry imaging (MSI) technique with minimum sample preparation, is promising. Previous applications of DESI-MSI in rock,^9,10^ meteorites,^11,12^ and asteroid samples^3,13^ have been constrained by low spatial resolution (200 μm). Consequently, previous works focused on limited scales and modalities and have not achieved spatial correlation between functional groups, organic molecular composition, and minerals.^3,4^

To overcome these limitations, we introduce the concept of cross-scale multimodal imaging, widely used in biological and medical research.^14,15^ A common combination involves vibrational spectroscopy imaging and mass spectrometry imaging, due to their complementary spatial resolution and molecular information.^14,15^ Compared to Matrix-Assisted Laser Desorption/Ionization (MALDI),^16^ DESI does not require matrix spraying,^17^ offering greater compatibility and flexibility with other analytical techniques when applied to extraterrestrial samples. Prior to the implementation of cross-scale multimodal imaging to the analysis of extraterrestrial samples, there are several analytical challenges that must be addressed, including sample preparation, appropriate workflow design, control of cross-contamination, and correction of matrix effect caused by complex minerals. Moreover, it is also essential to enhance the spatial resolution of DESI-MSI in order to establish clear associations with fine minerals (micron scale).

In this study, we propose a cross-scale multimodal imaging workflow for organic matter in extraterrestrial samples, while also addressing the mentioned analytical challenges. The workflow integrates vibrational spectroscopy imaging and mass spectrometry imaging (MSI) from millimeter to submicron scales, including five techniques: focal plane array-Fourier transform infrared spectroscopy (FPA-FTIR), optical photothermal infrared spectroscopy (O-PTIR), desorption electrospray ionization coupled quadrupole-time-of-flight mass spectrometry (DESI-Q-TOF/MS), time-of-flight secondary ion mass spectrometry (TOF-SIMS) coupled tandem MS, and nanoscale secondary ion mass spectrometry (NanoSIMS). This workflow was applied to the well-studied carbonaceous chondrite meteorite Murchison.^18^ Cross-scale multimodal imaging aims to simultaneously obtain mineral phases, functional groups, molecular compositions, and isotopic information on organic matter within the same sample region, thereby realizing spatially resolved correlations.

## Methods

### Sample Preparation

To obtain large, smooth cross sections of the Murchison meteorite without introducing organic contamination, resin embedding, slurry polishing, and solvent cleaning were avoided. A millimeter-sized sample was mounted on an SEM stub, and its top surface was cut, ground, and polished using a target preparation system (Leica EM TXP). The cross-section (Figures S1 and S2) was then further smoothed and decontaminated via broad argon ion milling (Leica EM TIC 3X). Argon ion milling parameters were changed alternatively in working to minimize heat load:^19,20^ including milling at 5 keV, 2 mA, and 20 min for four cycles, and milling at 1 keV, 1 mA, and 10 min for three cycles, all with an ion beam incidence angle of 3°. Argon ion milling was also employed post scanning electron microscopy and energy dispersive spectroscopy (SEM-EDS) analysis as well as after DESI-MSI, to mitigate organic contamination caused by the electron beam and solvent, respectively. Procedure blanks and solvent blanks need to be included to monitor potential contamination during sample preparation and analysis.

It is of paramount importance to achieve an ideally flat surface, as surface analysis techniques are highly sensitive to topography. The broad argon ion milling under mild conditions would not change the organic matter structure.^20^ Alternative techniques include argon ion cross-sectioning^21^ or the use of a plasma-focused ion beam (PFIB) to create large flat surfaces.^11,12^

### FPA-FTIR Measurement and Data Processing

Hyperspectral infrared imaging was performed in reflection mode using an FTIR microscope (Lumos II, Bruker Optics) equipped with an LN_2_-cooled 32 × 32 FPA detector. Subsequently, micro-ATR with germanium crystals was used to analyze a region near the edge of the sample (as indicated in Figure S2). Since ATR crystals damage the sample surface, only one area was analyzed. Adaptive K-means clustering was applied during the postprocessing of hyperspectral imaging data to produce a chemical phase image. Mineral phases were identified by the EDS and the mineral reflection spectra database (USGS Spectral Library Version 7).^22^ Reflectance spectra were transformed into absorbance spectra via Kramers–Kronig transformation (KKT), and the hydroxyl (O–H) absorption peak was integrated to create distribution maps. Both cluster analysis and KKT were implemented using OPUS software (v 8.8.4, Bruker Optics).

### O-PTIR Measurement and Data Processing

O-PTIR measurements were performed using a mIRage-LS system (Photothermal Spectroscopy Corp.) equipped with a quantum cascade laser (QCL) IR source covering ranges of 2990–2700 cm^–1^ and 1800–950 cm^–1^. A 532 nm probe laser with an output power of 200 mW at the aperture was attenuated to 1% to prevent laser-induced damage, resulting in less than 1 mW reaching the sample surface after accounting for internal optical losses. The IR laser power was adjusted between 10% and 80% based on the mineral’s photothermal response. No damage to the sample surface was observed postmeasurement. Raman spectra at select points were simultaneously acquired at the same locations as the O-PTIR spectra.

The O-PTIR technique is based on measuring collective photothermal effect changes in both the photothermal expansion and the localized refractive index changes upon absorption of infrared laser radiation at specific wavenumbers. The contribution from photothermal expansion is similar to that of the AFM-IR^23,24^ technique, except that the detection is based on the pinging of an AFM tip in contact, whereas a visible laser is utilized in the former. Therefore, the O-PTIR technique provides indirect subdiffraction-limited IR absorption measurements, but a submicron (about 500 nm) spatial resolution of the diffraction limit of the visible laser. The photothermal response is proportional to the absorption cross-section and thermal expansion coefficient but inversely proportional to thermal conductivity and heat capacity.^23^ Chemical absorption images were obtained by calculating the ratio between the absorption peak and reference wavenumber responses. The selection of the reference wavenumber should be away from transitions between QCL chips.

### DESI-Q-TOF/MS Measurement and Data Processing

DESI-MSI was conducted on a platform that included a DESI XS source (Waters), a Q-TOF/MS (SELECT SERIES Cyclic IMS, Waters), and a solvent delivery system (ACQUITY UPLC M-Class μBSM, Waters). Using the 95:5 MeOH/H_2_O as the solvent, the sample was imaged at positive ion mode with a 10 μm step size, with a solvent flow rate of 250 nL·min^–1^ and a scan speed of 40 μm/s. The flow rate was lower than the typical 1–2 μL·min^–1^ in regular DESI-MSI.^9,12^ A nanoEase BEH C18 Column (130 Å, 1.7 μm, 300 μm × 150 mm) was connected between the μBSM and sprayer to maintain backpressure at approximately 300 psi with a low flow rate of 250 nL·min^–1^. Leucine enkephalin (LE) at a concentration of 200 ng·mL^–1^ was added to the solvent as a Lock Mass, and Lock Mass calibration (*m*/*z* 556.2771 in positive mode) was conducted every 30 min. A total of 2500 ions were extracted to generate the MSI (HDI v1.8, Waters). The elemental composition of the ions was assigned via MassLynx software (v4.2, Waters). To minimize the impact of matrix effect, all ion intensities were normalized against the intensity of LE. The correction of matrix effect is further elaborated upon in the results section. For ease of visualization, normalized signal intensities were multiplied by 10,000.

Spatial segmentation (Table S2) was performed using uniform manifold approximation and projection (UMAP) combined with hierarchical density-based spatial clustering of applications with noise (HDBSCAN).^25^ The UMAP metric was based on Euclidean distance, and the HDBSCAN clustering method used the ″leaf″ criterion for cluster selection. Additionally, Ward’s hierarchical clustering^25^ was employed to analyze correlations among the 100 ions with the highest average intensity.

### TOF-SIMS Measurements

TOF-SIMS experiments were conducted using a Nano TOF3^+^ instrument (ULVAC-PHI, Inc.). TOF-SIMS (MS1) and TOF-SIMS/MS (MS2) measurements and imaging were both performed with 30 kV Bi_3_^+2^ as the primary ion and an ion beam current of 10.5 nA. Initially, secondary ion imaging of the region of interest (ROI) was performed in both positive and negative ion modes, accumulating 25 frames. The ROI was then sputtered with a gas cluster ion beam (GCIB) for 120 s, after which secondary ion imaging was repeated in both ion modes. Ions with higher *m*/*z* values and intensities were selected for collision-induced dissociation (CID) to generate product ions. Spectra and images of product ions were collected via MS2, while unselected ions were simultaneously collected via MS1.

### NanoSIMS Measurements

NanoSIMS measurements were performed on a NanoSIMS 50 L (Cameca). The sample was coated with Au as an electrically conductive layer before NanoSIMS analysis. Elemental and isotopic images were acquired in two different sessions at the same area (Figure S3). H, D and ^12^C were collected with a 10 pA Cs^+^ primary ion and a beam size of about 250–300 nm. ^12^C and ^13^C were collected with a beam current of 5 pA Cs^+^ primary ion and a beam size of about 150 nm. The coal reported in our previous study^26^ was used as the standard sample to calibrate instrumental mass fractionation (IMF) of H and C isotope.

Detailed information about the SEM-EDS, FPA-FTIR, DESI-Q-TOF/MS, TOF-SIMS, and NanoSIMS instrument setup and data processing can be found in the Supporting Information and Tables S1–S4.

## Results and Discussion

### FPA-FTIR: Global View of Mineral Phases with Hydroxyl Distribution

**Figure 1:**
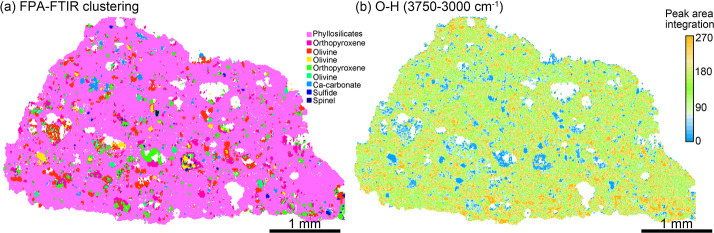
FPA-FTIR hyperspectral imaging of the Murchison meteorite. (a) The mineral phase map. Spectra of each phase are given in Figure S4. Olivine and orthopyroxene are distinguished as different phases in FPA-FTIR imaging due to differences in Mg and Fe content. (b) The hydroxyl distribution was created with the peak integration from 3750 to 3000 cm^–1^. The white voids are excessively low reflectivity or spectral artifacts caused by uneven topography inside the chondrules (Figures S1 and S2).

Murchison meteorite is classified as a CM2.5 type carbonaceous chondrite, primarily composed of matrix and chondrules.^18^ The matrix consists of fine-grained phyllosilicate minerals with other minerals like olivine (forsterite), sulfides (pentlandite, troilite), orthopyroxene, and Ca-carbonate occurring in granular within the interchondrule matrix or chondrules (Figures 1, S1 and S2). Fine-grained rims (FGRs)^27^ surround the chondrules (Figures S2 and S3) and share a consistent chemical composition with the phyllosilicates in the matrix,^28^ making them indistinguishable in phase maps (Figures 1a and S3). Therefore, when comparing FGRs and interchondrule matrix analysis results, the matrix effect due to differing chemical compositions can be minimized, but the influence from physical properties needs to be accounted. Since CM chondrites are complex breccias,^29^ significantly low-brightness regions can be observed in backscattered electron (BSE) and optical images (ROI 6 and 7, Figures S1 and S2), which may correspond to breccias with a higher degree of alteration.^29^

Reflection FPA-FTIR hyperspectral imaging combined with cluster analysis allows for the identification of different mineral phases. However, due to the diffraction limit, grains smaller than 10 μm cannot be resolved, and spectra may be distorted by signal mixing from fine minerals. FPA-FTIR imaging identified nine different mineral phases (Figure 1a). Variations in Mg and Fe content in olivine and orthopyroxene alter the Si–O absorption band shape and peak position in FTIR spectra (Figure S4),^30^ allowing these minerals to be subdivided into several phases.

A unique advantage of FPA-FTIR is its ability to provide both mineral phase identification and information on hydroxyl and organic functional groups. The FPA-FTIR and ATR-FTIR spectra showed consistent hydroxyl stretching (∼3550 cm^–1^) and bending vibrations (∼1624 cm^–1^) (Figure S5). No absorption bands corresponding to organic functional groups were observed in the FPA-FTIR spectra of Murchison meteorite. Hydroxyl groups, including Si-bonded O–H (3550 cm^–1^) and H-bonded O–H (3330 cm^–1^),^31^ are mainly concentrated in the phyllosilicates (Figure S4). Integration of the O–H peak area (3750–3000 cm^–1^) represents relative water content. The water content distribution appeared relatively uniform across the sample but showed a slight tendency to increase in the regions surrounding the chondrules.

FPA-FTIR imaging did not reveal organic functional groups in the Murchison meteorite, which could be attributed to factors such as the detection method (reflection or transmission), sensitivity, spectral signal-to-noise ratio, and the preserved form of organic matter in the meteorite. Currently, the preparation of large-area (hundreds of microns) ultrathin (a few microns) slices for transmission IR analysis remains challenging, as it risks disrupting the original mineralogical relationships. It is possible to achieve enhancements in sensitivity and SNR by using more powerful light sources, such as synchrotron radiation.^4,32^ Methyl, methylene, and carbonyl groups have been detected in the Ruygu asteroid with synchrotron-IR.^32^ Recently, the direct employment of QCLs can replace the traditional globar IR light source and QCL-IR imaging offers a speed increase of more than 100 times;^15^ however, its current capabilities are limited to the fingerprint region and do not extend to the stretching vibration bands of hydroxyl groups.

### DESI-MSI: The Big Picture of Organic Matter

As an in situ ionization mass spectrometry imaging technique, DESI-MSI inherently experiences matrix effect,^33^ a challenge also encountered in other widely used in situ mass spectrometry methods in geology, such as SIMS and Laser Ablation Inductively Coupled Plasma Mass Spectrometry (LA-ICP-MS). For example, differences in the chemical composition of white and gray matter in brain tissue can lead to significant variations in DESI signal yield for neurotransmitters.^34^ On rock surfaces, matrix effect are more complex and may arise from the following four factors: (1) chemical properties: the crystal structure and surface chemical characteristics of different minerals can affect solvent interactions, thereby influencing ion yield. (2) Physical properties: factors such as porosity, permeability, roughness, and conductivity all play a role; for instance, high porosity has been shown to reduce DESI response intensity.^9^ (3) Ion suppression or charge competition:^33^ signal intensities may not directly correlate with ion concentrations because ions are ionized simultaneously in situ. (4) Sample surface morphology: surface features like slopes and undulations can impact the geometric alignment among the sprayer, sample surface, and transfer inlet in DESI. Notably, the first three factors are likely to exert different influences on various ions.

**Figure 2:**
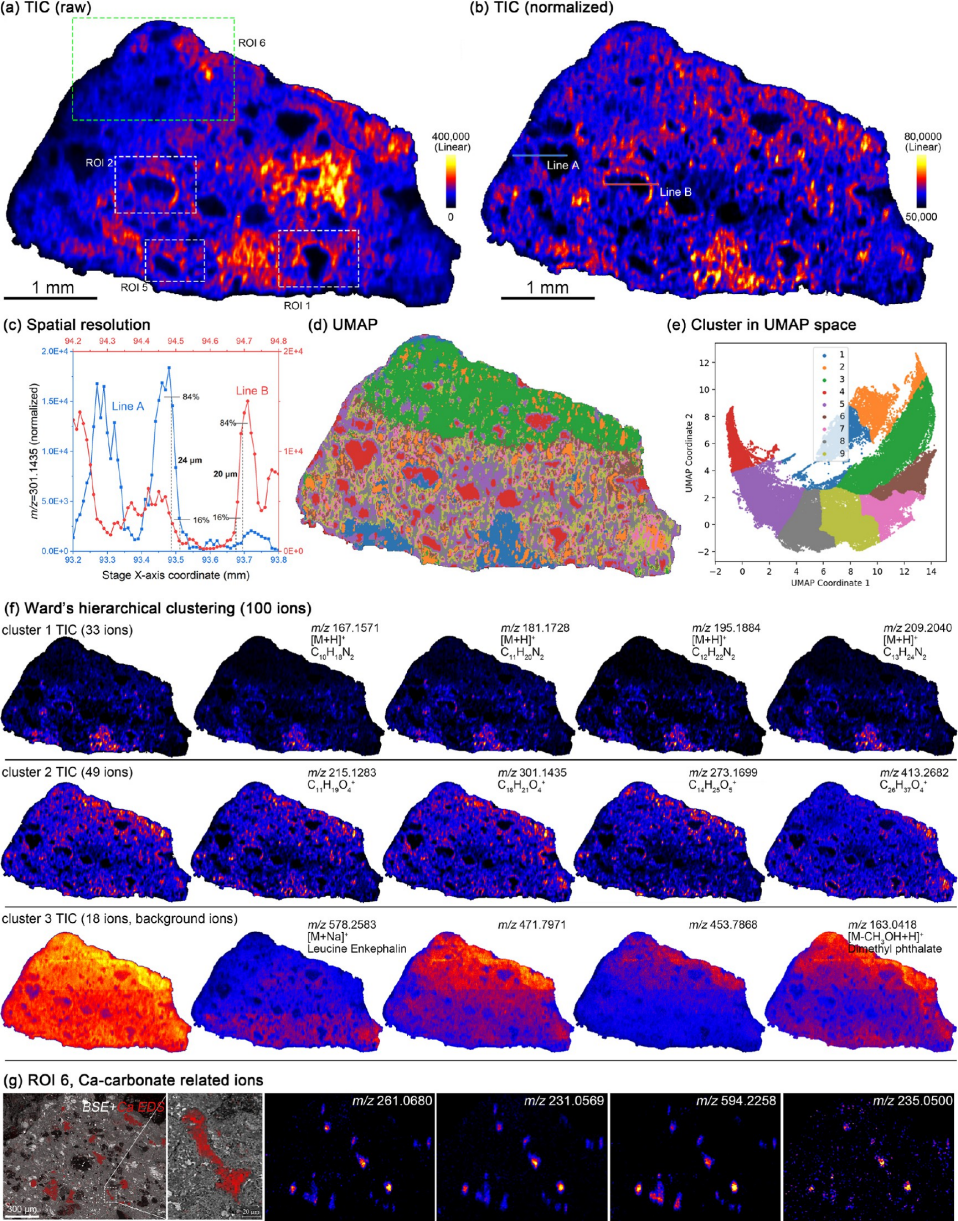
DESI-MSI analysis of the Murchison meteorite. (a) Raw total ion counts. (b) Normalized total ion counts, with each ion intensity normalized to the lock mass intensity. (c) Evaluation of spatial resolution, with the locations of Line A and Line B marked in (b). (d) Spatial segmentation results using UMAP and HDBSCAN. (e) Clusters in UMAP space. (f) Ward hierarchical clustering with the top four ions by intensity in each cluster. (g) Ions correlated with the Ca-carbonate in ROI 6.

In this study, 200 ng·mL^–1^ LE was added to the solvent as a lock mass to correct mass drift during analysis. Since the concentration of LE remains constant in the solvent, its distribution should theoretically remain uniform in the absence of matrix effect. However, variations in the LE signal response were observed (Figure S6), with particularly low signals on the left side of the sample and abnormally high signals in certain regions within the sample. Similarly, the overall signal response was lower in the left section of the sample (Figure 2a), confirming the influence of matrix effect. To address this, LE was used as an internal standard, and the intensities of all ions were normalized by dividing them by the LE signal to minimize matrix effect. This correction resulted in a more uniform intensity distribution across the sample and sharper, clearer feature boundaries (Figures S6, and 2b). As DESI-MSI in this study employs a nontargeted imaging approach, it is challenging to preselect internal standards with chemical structures similar to the target ions. Moreover, given that ionization efficiency varies among different ions due to charge competition, the use of LE for normalization cannot completely eliminate matrix effect. Future research should aim to further explore the factors contributing to matrix effect in DESI-MSI for rock and mineral samples. Nevertheless, at present, normalization using LE is deemed both appropriate and necessary to correct for these effects.

DESI-MSI achieved a spatial resolution of approximately 20 μm (Figure 2c), providing a global distribution of organic matter (Figure 2b). Total ion counts (TIC) distribution varies greatly (Figure 2b), especially with higher TIC in breccias having a higher degree of alteration (ROI 6 and 7 in Figure S2), and distinct rim structures with relatively high TIC were observed around chondrules, corresponding to the FGRs identified in Figures S2 and S3. The average TIC extracted from FGRs in the regions of interest (ROIs) was higher than that of the entire sample (Figure S7). In contrast, TIC was lower in olivine, orthopyroxene, and Ca-carbonate minerals (Figure S7b,c). Several ions showed positive spatial correlations with Ca-carbonate minerals (Figure 2g), while no such correlations were detected for olivine or orthopyroxene. These observations indicate that FGRs are enriched in organic matter but that certain organic molecules are more abundant in Ca-carbonate. Furthermore, DESI-MSI also revealed ions associated with Ca-carbonate particles approximately 20 μm in size (Figure 2g), further validating the spatial resolution of DESI imaging at 20 μm.

UMAP and HDMSCAN clustering spatially divide the entire sample into different clusters based on molecular species and abundance, reflecting the uneven distribution of organic matter composition and abundance within meteorites. Breccias with higher alteration degrees are grouped into a separate phase 1 in UMAP (Figure 2d,e). Spatial segmentation further identified FGRs and revealed compositional differences between the upper and lower sections of the sample. The results of spatial segmentation were corroborated by hierarchical clustering, which categorized the ions into three groups with distinct distribution patterns (Figure 2c). Cluster 1 ions were enriched in breccias with higher alteration degrees. Cluster 2 exhibited a more uniform distribution across the sample, while the diffuse distribution of Cluster 3 indicated that these ions likely originated from background components in the solvent, such as plasticizers.

A series of alkylated homologues of CHN and CHNO compounds can be identified in the Kendrick mass defect (KMD) plot^35^ (Figures S9 and S10), consistent with previous studies,^13,36^ and indicating the possible presence of amines, amides, amino acids, and N-containing heterocycles.^3,37^ Different CHN and CHNO series showed varied distributions (Figure S11). The distributions of alkylated homologues within a single CHN compound series were consistent (Figures S12, and S13). A series of –CH_2_ fragments appeared in the DESI-MS/MS spectrum of the precursor ion at *m*/*z* 273.1699 (Figure S8), which might be a kind of hydroxy dicarboxylic acid.^38,39^

### TOF-SIMS: Organic Matter in FGRs

**Figure 3:**
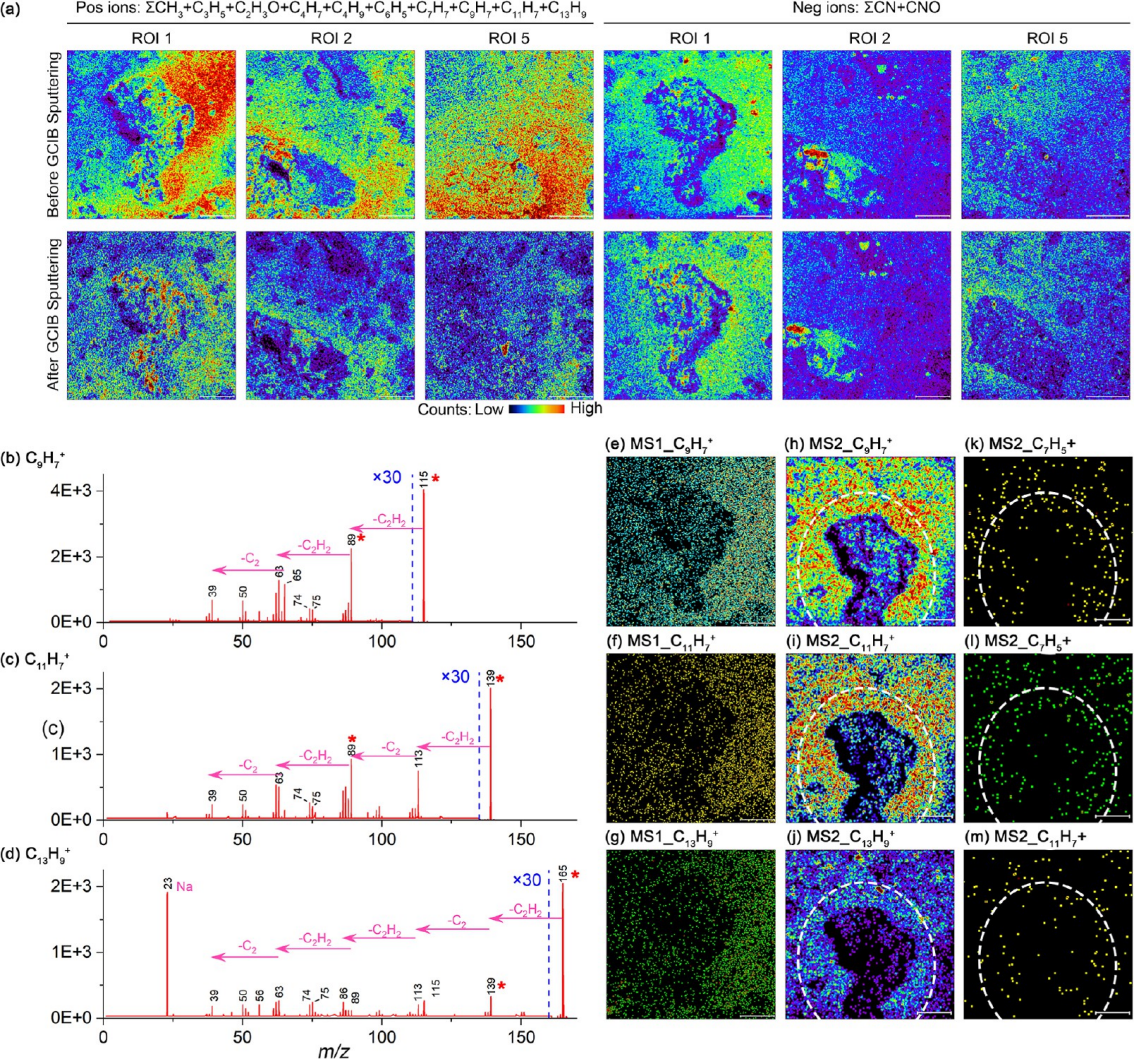
TOF-SIMS results of FGRs in the ROI. (a) MS1 ion imaging before and after GCIB sputtering (individual ion images in Figure S14). (b–d) MS2 spectrum. (e–g) MS1 imaging of precursor ions. (h–j) MS2 imaging of precursor ions. (k–m) MS2 imaging of product ions. The white dotted line marks the boundary between the FGR and the interchondrule matrix. All scale bars are 100 μm.

A series of fragment ions related to alkyl (e.g., C_3_H_5_^+^, C_4_H_7_^+^, C_5_H_7_^+^, C_6_H_9_^+^) and phenyl functional groups (e.g., C_6_H_5_^+^, C_7_H_7_^+^) were collected in the range of *m*/*z* 0–200 in the MS1 spectrum in positive mode (Figure S15). The GCIB effectively minimizes damage to the organic molecular structure,^40^ allowing for the removal of potential organic contaminants on the sample’s surface without significantly compromising the secondary ionization yield of organic components within the meteorite. The enrichment of organic signals in the FGRs remained evident (Figures 3a, and S14), indicating that the observed enrichment was not due to contamination. The MS1 spectrum aligns with previous TOF-SIMS analyses of meteorites.^41,42^ The elemental compositions of ions with *m*/*z* 98–300 Da and relative intensities above 1% (normalized to *m*/*z* 115.0503) were assigned to carbon and hydrogen (Figure S16). Identified ions included polycyclic aromatic hydrocarbons (PAHs) such as naphthalene (C_10_H_8_), acenaphthylene (C_12_H_8_), phenanthrene or anthracene (C_14_H_10_), and pyrene (C_16_H_10_). The TOF-SIMS mass spectrum differs markedly from that of DESI-MSI,^13^ likely reflecting the influence of ionization methods, as insoluble organic matter with complex aromatic structures constitutes most of the organic content in meteorites.^43^

Organic ion fragments in the FGRs—including alkyl and phenyl fragments, as well as CN and CNO ions detected in negative ion mode—exhibited higher intensities compared to the interchondrule matrix (Figures 3a, and S14). Given the chemical similarity between FGRs and matrix, potential matrix effect in TOF-SIMS can be excluded. Notably, during sample preparation, organic matter accumulated in pits within chondrules, leading to increased TOF-SIMS signals in these areas (Figure 3a). TOF-SIMS analysis thus confirms the organic matter tend to enrich in FGRs, consistent with DESI-MSI findings.

While differences in ion intensity were observed across the left and right sides in the MS1 imaging (Figure 3), the intensity distribution of precursor ions in MS2 was relatively uniform. A distinct annular pattern with high ion intensity around chondrules—corresponding to the FGRs—was evident, highlighting the ability of MS2 to reduce background interference and provide a more reliable distribution of target molecules.^44^ The product ions in MS2 also showed enrichment in FGR.

### O-PTIR: Organic Matter in Ca-Carbonate

Olivine and Ca-carbonate exhibit lower O-PTIR signal responses compared to phyllosilicates (Figure S17) due to their higher thermal conductivity and heat capacity.^45−47^ To mitigate this, the IR image is normalized using a reference wavenumber with sufficient signal strength to remove nonabsorption-related contributions.^48,49^

**Figure 4:**
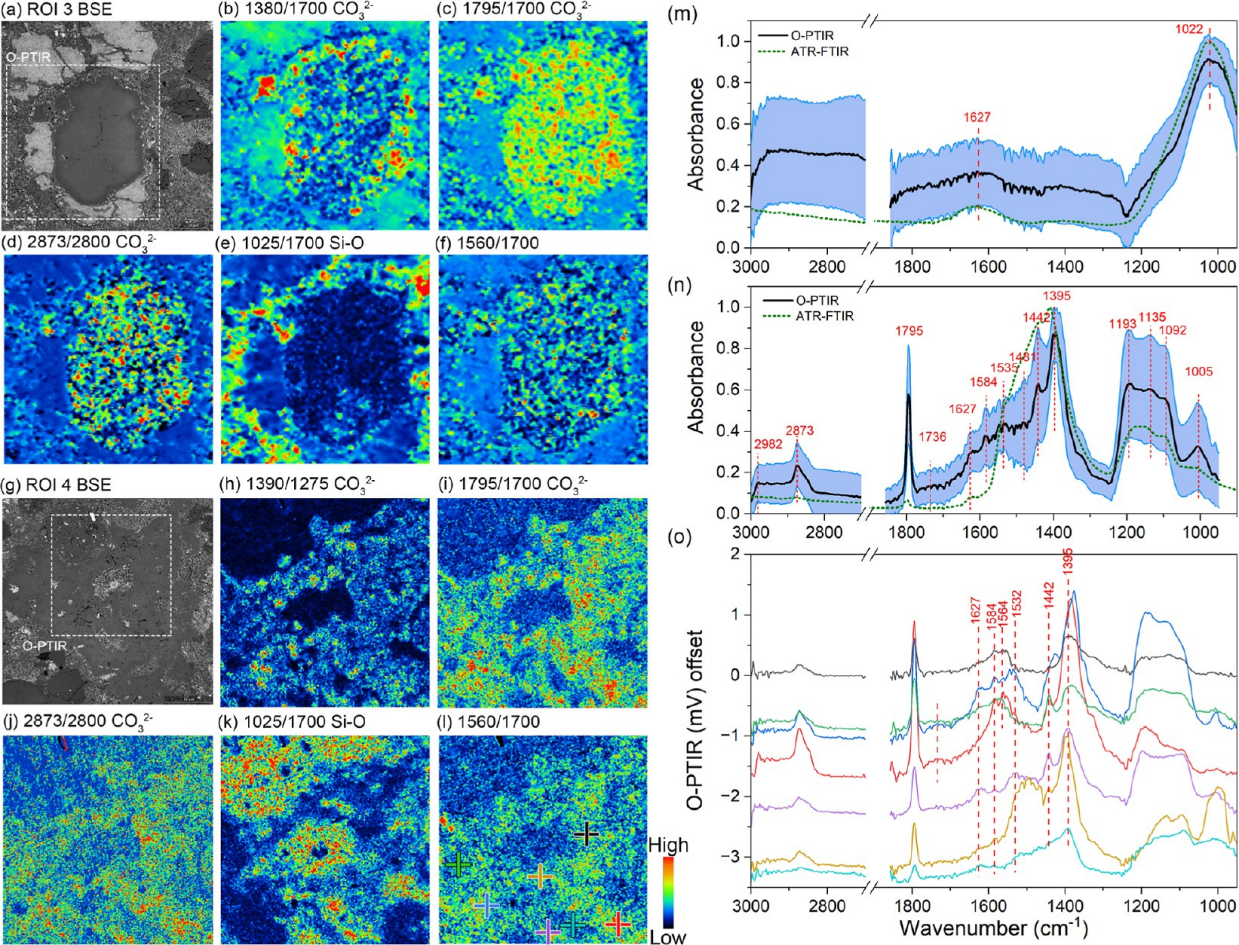
O-PTIR results of ROI3 and 4. (a–l) O-PTIR images of Ca-carbonate in ROI 4 and ROI 3. Each image size is 80 × 80 μm. (m,n) The average O-PTIR spectra of Ca-carbonate and phyllosilicates in interchondrule matrix. (o) O-PTIR spectra of Ca-carbonate. The location for each spectrum is marked with a cross in (I).

Subtle differences between O-PTIR spectra of the same mineral (Figure S18) reflect that O-PTIR is affected by variability in mineral chemical composition at the submicron scale. No absorption peaks of organic functional groups were detected in the O-PTIR spectrum of phyllosilicates (Figure 4m). The broad peak at 1627 cm^–1^ is attributed to the bending vibration of H–OH. The nested distribution of calcium carbonate and phyllosilicates can be identified (Figure 4e,k). The two peaks located at 2982 and 2873 cm^–1^ in Ca-carbonate are assigned to the combination bands or overtones of carbonate,^50−52^ rather than the methyl or methylene that are generally located between 3000 and 2800 cm^–1^. Spectra of Ca-carbonate obtained from different locations exhibit variations (Figure 4o). Peak shifts around 1400 cm^–1^ can indicate the heterogeneity of the internal chemical composition and crystal structure of Ca-carbonate. Due to the possibilities of combination bands or overtones in Ca-carbonate,^50−52^ the assignment of peaks at 1600–1500 cm^–1^ requires greater caution. The peak at 1627 cm^–1^ should also be attributed to the bending vibration of H–OH, while 1584 cm^–1^ may indicate the presence of C=C bonds in the ring. Simultaneous Raman spectra collected at the same locations (Figure S18) show the characteristic D-band and G-band of amorphous organic matter in both Ca-carbonate and phyllosilicates,^53^ whereas olivine shows no such signatures. These observations are consistent with DESI-MSI results, which reveal the absence of organic matter in olivine.

Submicron IR (O-PTIR) has been numerously demonstrated in identifying organic matters in inorganic substrates, such as fluid inclusions in oil exploration,^54−56^ biofilms in deep sea rocks,^57,58^ archeological discoveries,^59^ unknown organic matter in microelectronics,^60,61^ and analysis of carbonate-based paints in cultural heritage.^62,63^ Organic matter in nanoscale spatial resolution (<100 nm) can be further investigated via AFM-IR^4,6^ and infrared-scattering-type scanning near-field optical microscopy (IR-sSNOM),^7^ but these ultrahigh resolution techniques will require strictly smooth surface preparations to enable AFM-based topological scanning.^64,65^

### NanoSIMS: Elements and Isotopes

**Figure 5:**
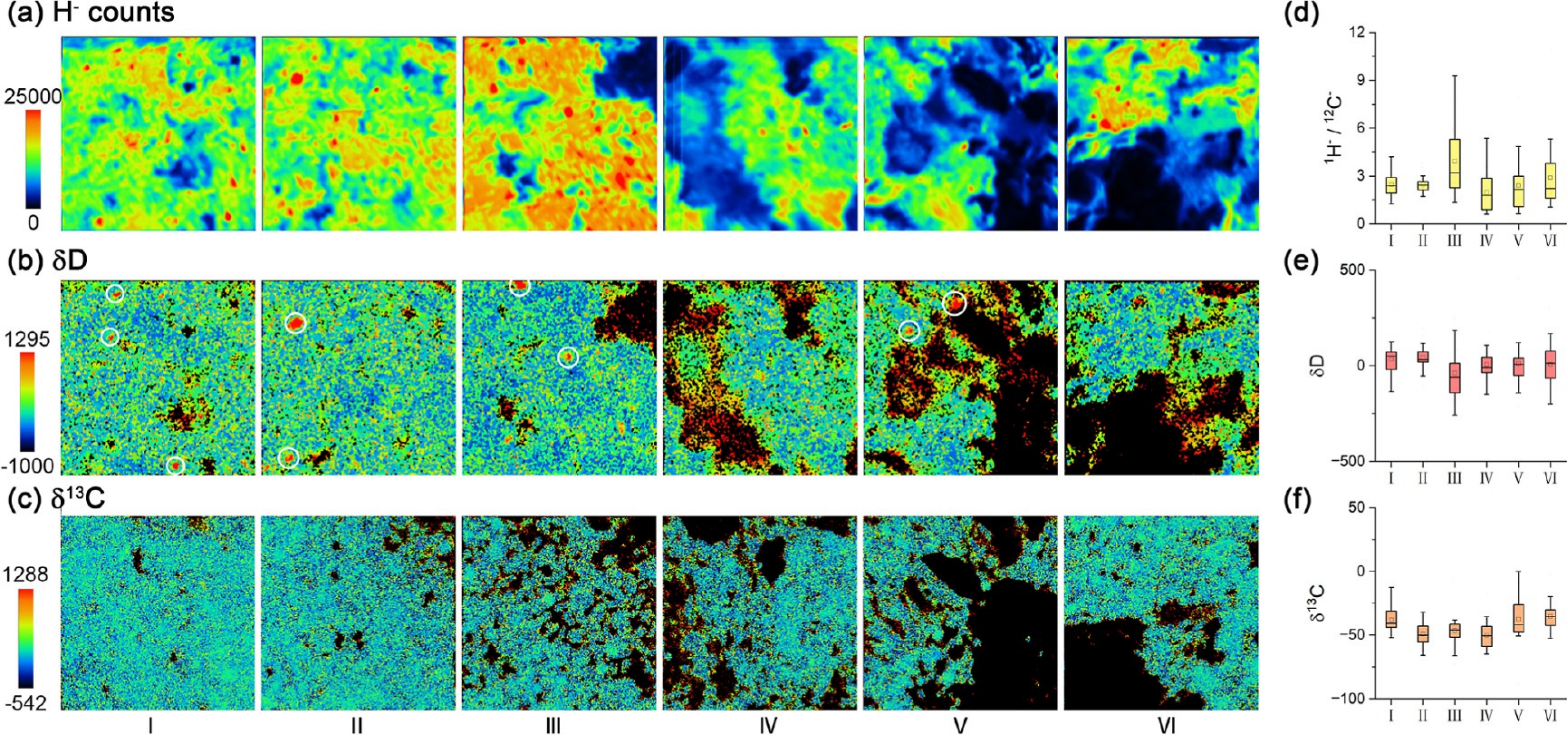
NanoSIMS imaging of FGR in ROI 2 (a–c) and the distributions of the ^1^H^–^/^12^C^–^ counts ratio, δD, and δ^13^C value from (I) to (VI) (d–f). Each image size is 20 × 20 μm. The location of the NanoSIMS image is marked in Figure S3. (I) to (VI) correspond to the locations from the inner side of the FGR to the outside of the interchondrule matrix. White circle mark D hot spots in (b). (d–f) Reflect the median, average, and distribution of multiple regions in each imaging of (a–c).

No systematic deviation in the ^1^H^–^/^12^C^–^ counts ratio was observed across the FGR and interchondrule matrix (Figure 5a,d). Similarly, δD and δ^13^C values showed no notable changes (Figure 5b,c,e,f), aligning with previous isotopic compositions reported in CM-group carbonaceous chondrites.^3,4^ Despite the enrichment of organic matter in FGRs revealed by DESI-MSI and TOF-SIMS, no isotopic difference was detected, suggesting a common origin for the organic matter in FGRs and interchondrule matrix. Additionally, D-rich hotspots (>4SD)^66^ with δD values ranging from 1000 to 4000‰ were identified (Figure 5b), potentially related to isotopic fractionation at low temperatures in the nebula.^4^ Previous studies have reported a difference in the abundance of presolar grains between FGRs and the interchondrule matrix.^66^ These hotspots are more prevalent in FGRs (7 items) than in the matrix (2 items), but further analysis is needed to confirm their distribution statistically.

### Implications, Challenges and Prospects

Multimodal imaging represents a paradigm shift in research methodology with regard to understanding the formation and evolution of complex organic matter. By integrating multiple imaging techniques within the primitive mineral texture, it provides a comprehensive view of the occurrence, composition, structure, and isotopic signatures of organic matter within the same sample region, surpassing the capabilities of individual techniques. While cross-scale multimodal imaging is commonly used in biological and medical fields, this study demonstrates its value in geosciences as well.

The ability to establish spatial associations between organic matter and mineral phases or structures (e.g., FGRs) is a key advantage of this approach. It allows for cross-method corroboration, providing a clearer picture of the complex relationships between organic matter and minerals. This technique is especially valuable in studying extraterrestrial organic matter and its environmental correlations. In this study, we present the first evidence of organic matter enrichment in FGRs, offering new chances for studying the origin and formation mechanisms of organic matter in meteorites, a topic that remains under active debate.^27,28^ The application of cross-scale multimodal imaging holds great promise for the analysis of future Martian samples and the search for organic matter on Mars. Furthermore, this workflow can be extended to a wide range of applications across various fields, including forensic science, petroleum exploration, paleontology, paleoanthropology, and archeology.

The availability of this workflow is largely attributed to the enhanced spatial resolution of DESI-MSI, which has reached 20 μm—a notable improvement over previous applications on rock,^9,10^ meteorites,^11,12^ and asteroid samples.^3,13^ Further advancements in spatial resolution are expected in the future. This improvement enables the integration and fusion of DESI-MSI data with IR, BSE, and EDS imaging, as well as the establishment of spatial associations with techniques such as TOF-SIMS, O-PTIR, and NanoSIMS. TOF-SIMS, with higher spatial resolution and a different ionization mechanism, provide a complement to DESI-MSI results. Tandem mass spectrometry, whether coupled with DESI-MSI or TOF-SIMS, aids in analyzing macromolecular structures in situ,^44^ while NanoSIMS provides isotopic composition data.

The proposed workflow follows a “top-down” procedure, as outlined in the methods section, which simplifies the process of narrowing down and targeting specific regions of interest. Ion milling is employed after BSE-EDS and DESI-MSI imaging to clean the sample surface. However, one challenge remains: the penetration depth of the DESI solvent on the surface of the rock is not yet fully understood and may vary depending on the physical properties of the minerals, such as their porosity or surface texture. Additionally, matrix effect need to be carefully considered. Without appropriate correction, matrix effect have the potential to give rise to misinterpretations of the results, as observed in techniques such as O-PTIR and DESI-MSI.

For biological tissue samples, hematoxylin and eosin (H&E) stains are widely used as substrate maps to link MSI and vibrational spectroscopy imaging.^15^ For geological samples, we suggest using BSE images as base maps to facilitate colocalization between multimodal imaging techniques. Mineral or feature boundaries can be extracted from BSE images, or mineral phases identified via EDS can be used to segment and extract organic molecules or functional groups associated with each mineral phase in MSI and IR imaging. The implementation of this approach will require the development of specialized data processing software to enable the efficient extraction and correlation of information from different modalities. On this basis, correlations between various imaging data sets can be explored, such as the relationship between water content (Figure 1) and molecular abundance (Figure 2).

Since FPA-FTIR and DESI-MSI generally offer comparable spatial resolution and scale, achieving spatial correlation between these techniques and BSE-EDS is relatively straightforward. However, when integrating TOF-SIMS, O-PTIR, and NanoSIMS data, careful attention must be paid to the differences in resolution and scale. In future work, the focus could shift from the single-modal spatial clustering used in this study to feature fusion across different modalities. This would enable the generation of a unified data set, allowing for more robust downstream spatial segmentation and clustering, and ultimately enhancing the ability to resolve complex sample structures.

## Conclusion

This study presents a novel cross-scale multimodal imaging workflow for extraterrestrial samples, integrating multiple analytical techniques to comprehensively characterize organic matter and its association with inorganic minerals. The enhanced spatial resolution of DESI-MSI (20 μm) facilitates its integration with other imaging techniques. This approach allowed us to observe differences in the composition and distribution of organic matter in interchondrule matrix, Ca-carbonate minerals, and fine-grained rims (FGRs) around chondrules, which had not previously been identified. The cross-scale multimodal imaging approach, combining mass spectrometry imaging and vibrational spectroscopy, represents a shift in the research paradigm and can be applied to other fields that require detailed spatial resolved analysis of complex materials.

## Supporting Information Available

Four tables and 18 figures. Divided into 6 parts: (1) A detailed analysis setting of FPA-FTIR, DESI-MSI, TOF-SIMS, and NanoSIMS (Tables S1–S4). (2) Optical image, BSE, EDS, and phase map of the Murchison meteorite (Figures S1–S3). (3) FPA-FTIR and ATR-FTIR spectra (Figures S4, and S5). (4) The results of DESI-MSI (Figures S6–S13). (5) TOF-SIMS imaging of FGRs and TOF-SIMS spectrum (Figures S14–S16). (6) O-PTIR spectrum (Figures S17, and S18) (PDF)
