# Supplementary material for: Cross-Scale Multimodal Imaging for Organic Matter in Extraterrestrial Samples
Source: Anal Chem. 2025 Mar 18;97(15):8258–67. doi: 10.1021/acs.analchem.4c05804 (PMC12019776; doi:10.1021/acs.analchem.4c05804)
Supplement: Supplementary file 1 — ac4c05804_si_001.pdf [file ac4c05804_si_001.pdf]

Cross-scale multimodal imaging for organic matter in extraterrestrial samples

Mingtan Dong [a,b], Wei Yang [a], Jialong Hao [a, *], Xiaofei Jia [c], Ou Yang [d], Michael K. F. Lo [e], Bobo Cao [f], Sen Hu [a], Yangting Lin [a]

[a] *Key Laboratory of Earth and Planetary Physics, Institute of Geology and Geophysics, Chinese Academy of Sciences, Beijing, 100029, China*

[b] *University of Chinese Academy of Sciences, Beijing, 100049, China*

[c] *Waters Corporation, Beijing,100076, China*

[d] *ULVAC-PHI Instrument Co. Ltd., Nanjing, 211102, China*

[e] *Photothermal Spectroscopy Corp., 325 Chapala St., Santa Barbara, CA 93101, USA*

[f] *Department of Chemistry, Tsinghua University, Beijing,100084, China*

* Email: sean_hao@mail.iggcas.ac.cn

Content

Methods **3**

SEM-EDS 3

FTIR measurements setting 3

DESI-MSI condition 4

TOF-SIMS measurements 5

Nano-SIMS measurements 6

Results **7**

Figure S1. Optical image of the cross-section of the Murchison meteorite. 7

Figure S2. BSE image of the cross-section of the Murchison meteorite. 7

Figure S3. BSE, EDS mapping, and phase map of ROIs. ROI is marked in Figure S2. 8

Figure S4. Average reflection FPA-FTIR spectra of clusters (a) and spectra after KKT (b). 9

Figure S5. Comparison of the FPA-FTIR reflection spectrum after KKT to the ATR-FTIR spectrum. 10

Figure S6. DESI-MSI of ions before and after normalization with lock mass. 11

Figure S7. Average DESI-Q-TOF/MS spectra of the entire sample and different minerals. 12

Figure S8. DESI-MS/MS spectrum of *m/z* 273.1699. 13

Figure S9. Kendrick mass defect (KMD) plot of DESI-Q-TOF/MS spectra. 14

Figure S10. Kendrick mass defect (KMD) plot of DESI-Q-TOF/MS spectra and element composition assignments. 15

Figure S11. Spatial distribution of CHN and CHNO compounds. 16

Figure S12. Spatial distribution of $C_nH_{2n-1}N$ alkylated homologues. 17

Figure S13. Spatial distribution of $C_nH_{2n-2}N_2$ alkylated homologues. 18

Figure S14. TOF-SIMS imaging (a, b, c) of FGRs in the ROI before and after GCIB sputtering. 19

Figure S15. TOF-SIMS spectrum in positive mode. 20

Figure S16. The C and H numbers of CH compounds in TOF-SIMS. 20

Figure S17. O-PTIR response for the different minerals under the same measurement conditions. 21

Figure S18. O-PTIR spectra and Raman spectra of calcite, olivine and phyllosilicate

References **23**

Methods

SEM-EDS

To avoid conductive coatings incompatible with subsequent MS and IR analyses, we used a low-vacuum desktop scanning electron microscopy (SEM) (Phenom XL G2) with integrated Energy Dispersive Spectroscopy (EDS), operating at 60 Pa and 15 kV. Large-area backscattered electron (BSE) and EDS mosaics of the entire sample were acquired using MAPS 3.29 software (Thermo Fisher). The pixel resolution of EDS mapping was 3 μm, and the Dwell time for each EDS point was 1 ms. Phase mapping was performed based on EDS data using the Phenom User Interface software (v2.0.0). Potential carbon contamination of the sample surface may occur due to the electron beam[1]. The sample surface was cleaned via an argon ion milling system after BSE-EDS analysis.

FTIR measurements setting

Table S1. FTIR measurements setting

FPA-FTIR detector	LN_2-cooled 32×32 FPA detector
Accumulation	32 scans
Detector pixel binning	2×2
Pixel size after binning	10 μm
Spectral resolution	4 cm^{-1}
Spectral range	4000-750 cm^{-1}
Background	Gold mirror
Post-process	Kramers–Kronig transformation (KKT), OPUS v8.8.4
	Adaptive K-means clustering, OPUS v8.8.4
ATR-FTIR detector	LN_2 cooled-MCT detector
Accumulation	64 scans
Spectral resolution	4 cm^{-1}
Spectral range	4000-600 cm^{-1}
Background	Air
Post-process	ATR correction, OPUS v8.8.4

DESI-MSI condition

Table S2. DESI-MSI condition

DESI source	DESI XS
Sprayer working distance	~ 1 mm above the sample surface
Sprayer incident angle	70°
Capillary voltage	0.8 kV for positive mode
Cone voltage	40 V
Source temperature	100 °C
Heated transfer line temperature	200 °C
Nebulizer gas	N_2, 1.3 bar
Solvent delivery	ACQUITY UPLC M-Class μBSM
Solvent	95:5 MeOH:H_2O, 0.1% formic acid, 200 ng/mL Leucine Enkephalin (m/z 556.2771 for positive mode)
Column	C18 column (130Å, 1.7 μm, 300 μm X 150 mm, Waters nanoEase M/Z Peptide BEH)
Solvent flow rate	250 nL/min
Stage speed	40 μm/sec
Sampling step size	10 μm
Mass spectrometer	SELECT SERIES Cyclic IMS
TOF mode	V mode
Mass range	100-600 Da
CID voltage	6-40 V
Data processing	
MSI	High Definition Imaging v1.8
Elemental composition analysis	MassLynx v4.2
Spatial segmentation and cluster analysis	MSI Segmentation v2.1.
UMAP and HDNSCAN parameters	
UMAP n_components	2
UMAP n_neighbors	10
UMAP Min_dist	0.025
UMAP metric	Euclidean
UMAP random_state	0
HDBSCAN min_samples [#pixels]	30
HDBSCAN cluster selection method	leaf

TOF-SIMS measurements

Table S3. TOF-SIMS condition

Primary ions	LMIG (Bi_3^{++})
Beam voltage	30 kV
Emission current	0.5 μA
Beam current	10.5 nA (DC)
Analysis Mode	High mass resolution mode (Bunched Mode)
Mass resolution	m/z=104 at standard sample (PET) >10,000
	m/z=91 $C_7H_7^+$ at meteorite > 6,000
Raster size	400 μm x 400 μm/500 μm x 500 μm
MS1 spectrometer	TRIFT (Triple Ion Focusing Time of Flight) analyzer
Ions scans mode	Positive and Negative mode
Mass range	2-1850 Da
Raster pixel	512 x 512
Acquire time	25 Frames (~ 14 min)
Flight length	2 m
Mass calibration	CH_3^+, $C_2H_3^+$ and $C_3H_5^+$ for positive mode
	CH^-, C_2H^- and C_4H^- for negative mode
MS2 spectrometer	TOF (Time of Flight) analyzer
Ions scans mode	Positive mode
Mass range	2-1850 Da
Raster pixel	256 x 256
Acquire time	200 Frames (~26 min)
Pressure of CID	0.05 Mpa
Flight length	1 m
Sputtering ions	Gas cluster ions beam (Ar_{2500}^+)
Beam voltage	10 kV
Beam current	10 nA
Sputtering area	900 μm x 900 μm
Sputtering time	2 min
Neutralize Setting	E-Neut+I-Neut
Data processing	TOF-DR 3.4.0.7

Nano-SIMS measurements

Table S4. Nano-SIMS condition

		**Session 1**	**Session 2**
Primary column setup	Primary ions	Cs^+	Cs^+
	Beam voltage	16 kV	16 kV
	Pre-sputtering current	300 pA	300 pA
	Pre-sputtering area	25 µm	25 µm
	Pre-sputtering time	10 min	1 min
	Analysis beam current	10 pA	5 pA
	Beam size	250-300 nm	~150 nm
	Raster size	20 µm	20 µm
	Raster pixel	256 × 256	512 × 512
	Dwell time	7.63 ms/pixel	3.82 ms/pixel
	Frames	14	7
Mass spectrometers setup	Secondary ion species	H, D, ^{12}C, ^{18}O	^{12}C, ^{13}C, ^{18}O, $^{12}C^{14}N$, $^{12}C^{15}N$
	Detector	EM×4	EM×5
	Entrance slit width	50 µm	20 µm
	Aperture slit	NA	350 µm
	Energy slit	NA	20%
	Mass resolution (Cameca definition)	~3000	~9000
Other setups	Analysis chamber vacuum	2E-10 mbar	2E-10 mbar
	Neutralize	E-gun	E-gun
Post-processing	Data processing software	ImageJ with Open MIMS plugin	
	Isotopic ratios calculation	$\delta R(‰) = (R_{sample}/R_{standard} -1) \times 1000$<br>R standard:<br>Standard Mean Ocean Water (SMOW: D/H= 155.76×10^{-6})<br>ienna Pee Dee Belemnite (VPDB:$^{13}C/^{12}C$ = 0.0112372)	
	Coal Standard sample[2]	δD= -147±1.1 (1SD)<br>$\delta^{13}C_{VPDB}$= -23.45±0.89 (1SD)	

Results

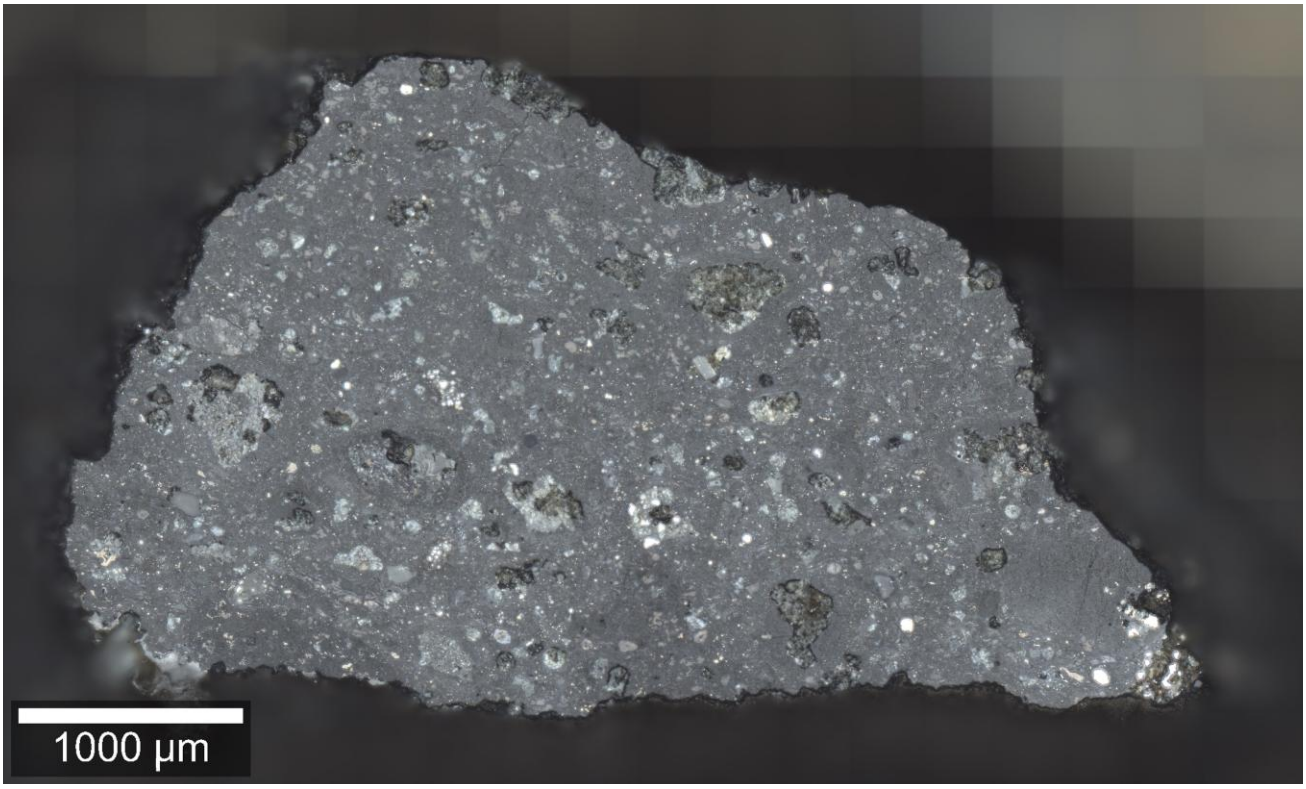

Figure S1. Optical image of the cross-section of the Murchison meteorite.

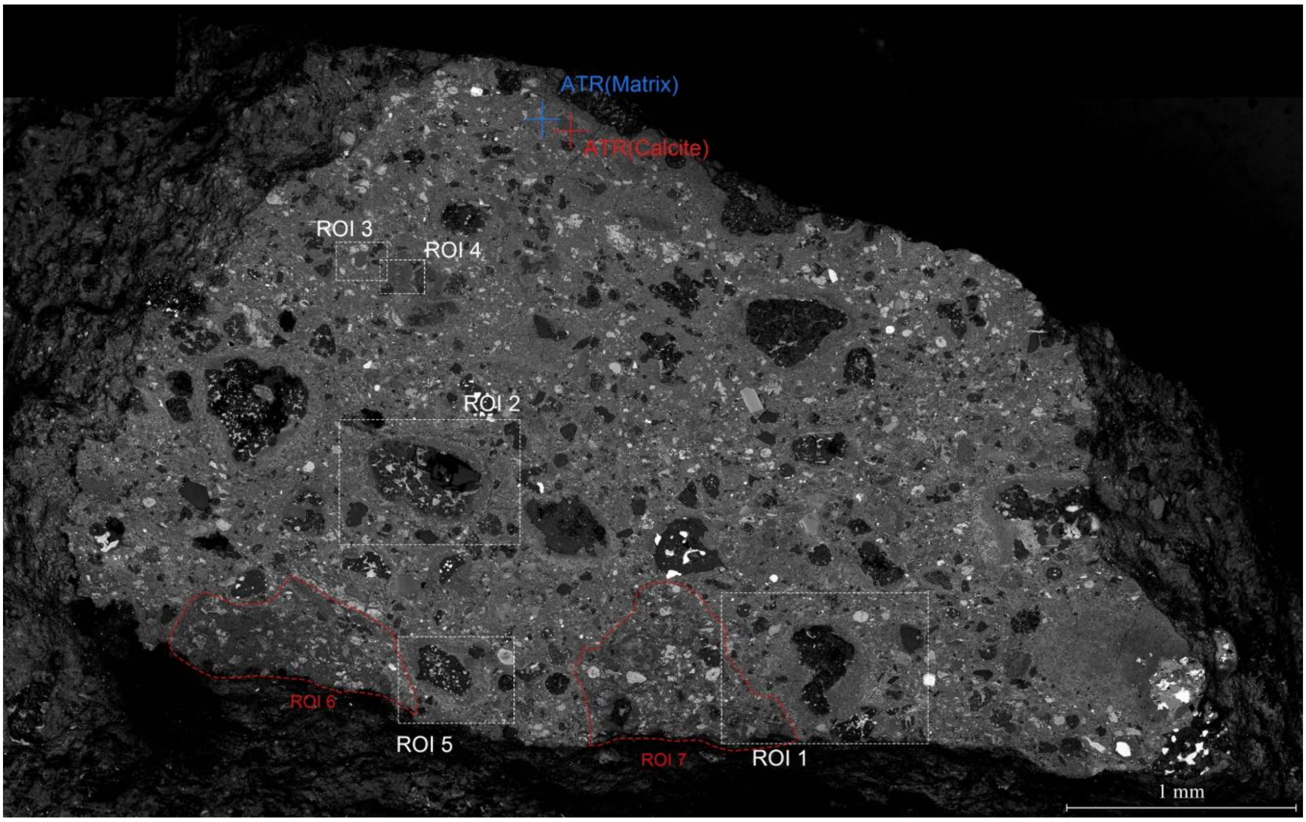

Figure S2. BSE image of the cross-section of the Murchison meteorite.

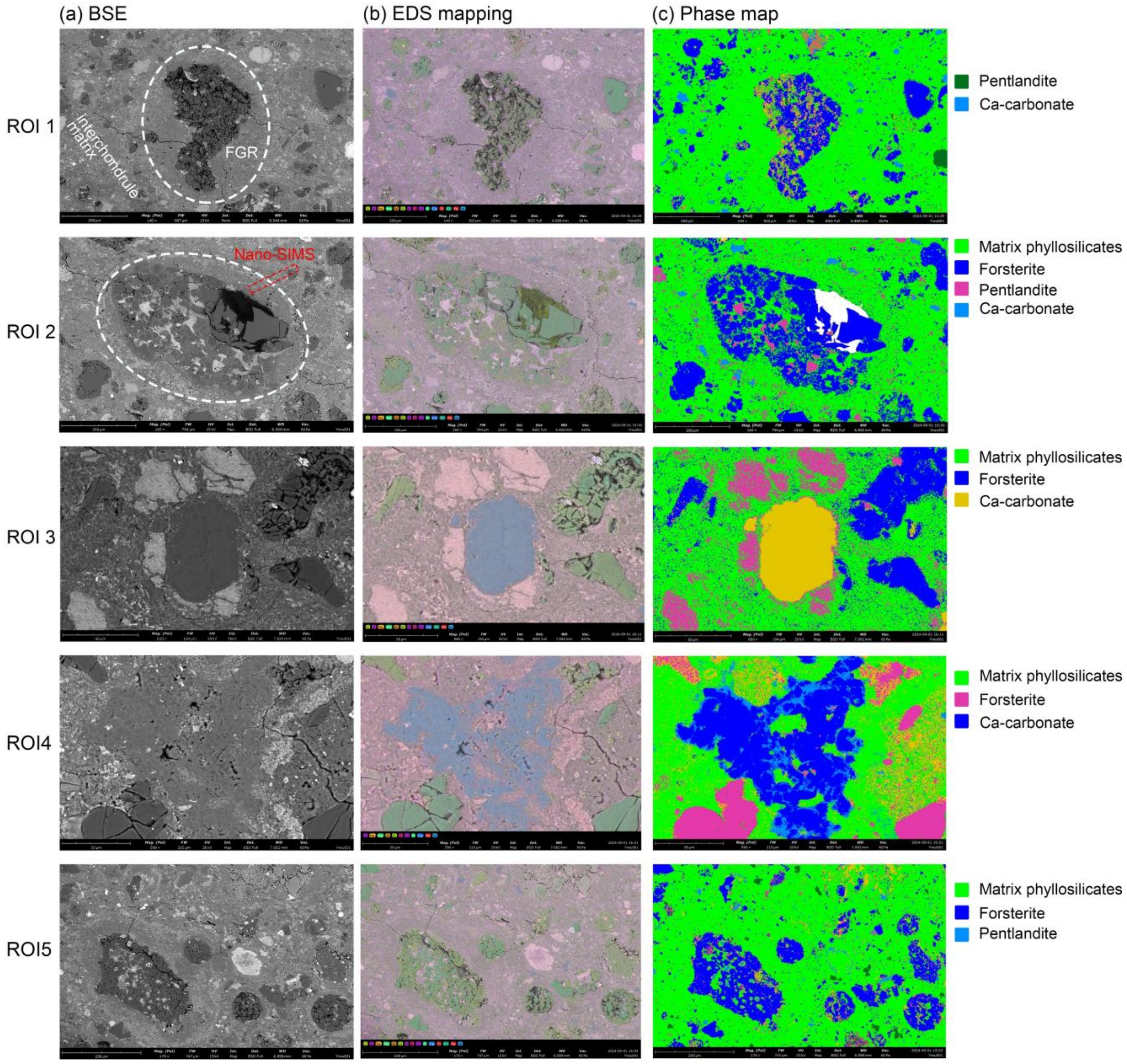

Figure S3. BSE, EDS mapping, and phase map of ROIs. ROI is marked in Figure S2.

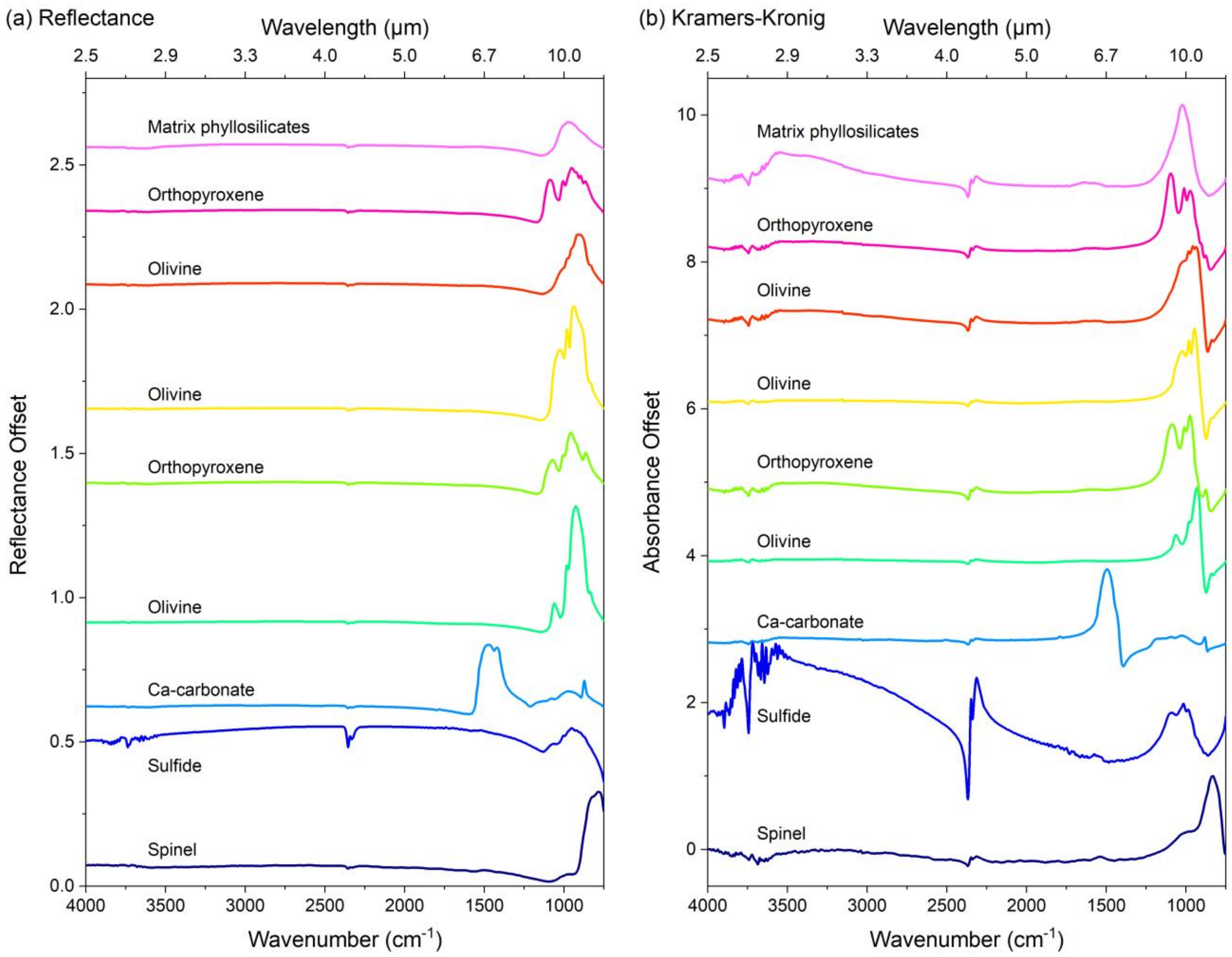

Figure S4. Average reflection FPA-FTIR spectra of clusters (a) and spectra after KKT (b). Because of the differences in Mg and Fe contents, Olivine and orthopyroxene have been divided into different phases in FPA-FTIR imaging. KKT may not produce correct transformation results for bands with large absorption indices (k>1), such as those of carbonates and sulfates[3]. The diffuse reflection component in the reflection spectrum also affects the accuracy of the KKT results. The reflection FPA-FTIR spectrum of the matrix phyllosilicates was consistent with the ATR-FTIR spectrum (Figure S5b), but there was an 80 cm^{-1} gap in the CO_3^{2-} absorption peak of Ca-carbonate between the reflection FPA-FTIR spectrum and the ATR-FTIR spectrum (Figure S5a).

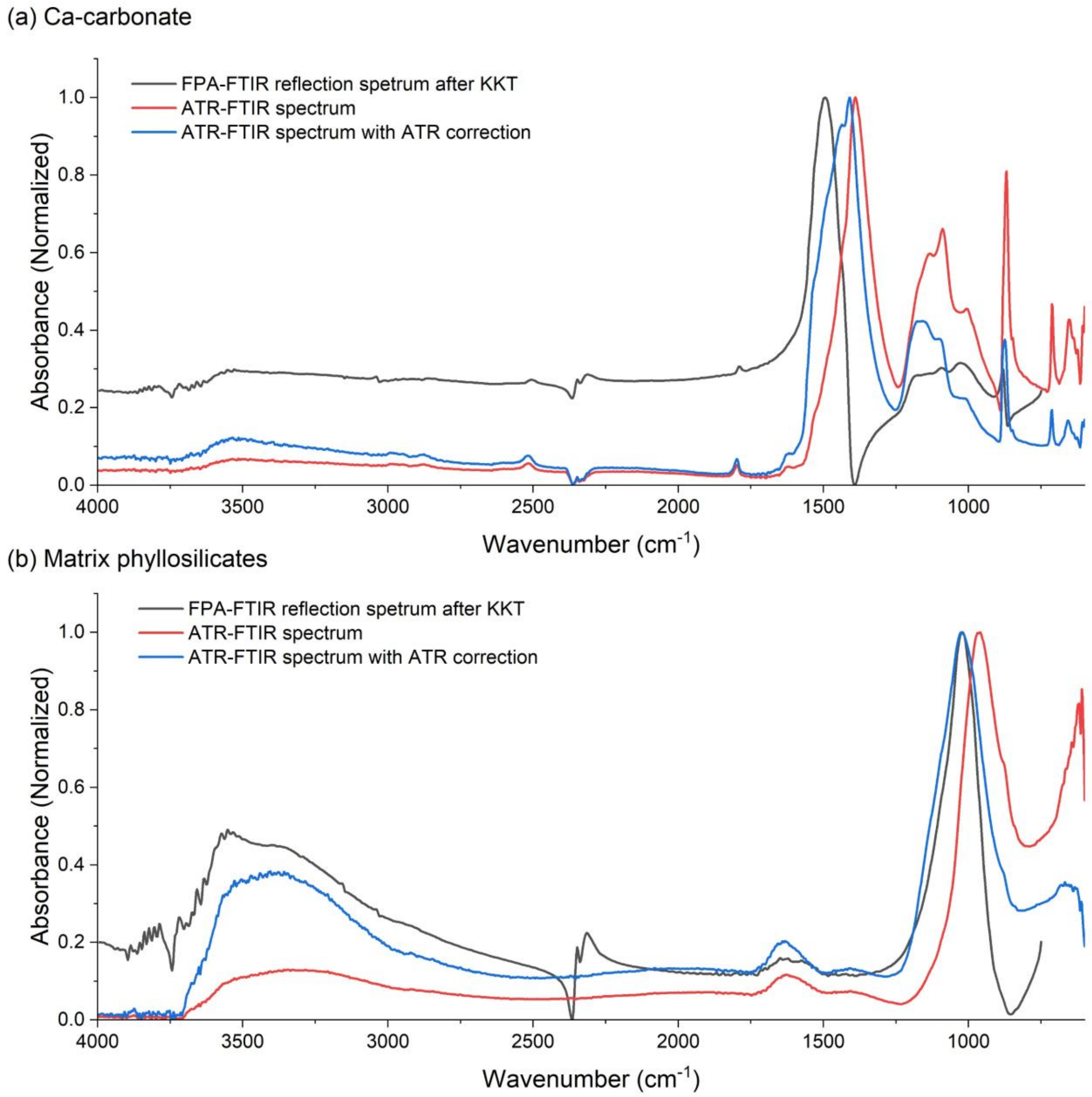

Figure S5. Comparison of the FPA-FTIR reflection spectrum after KKT to the ATR-FTIR spectrum.

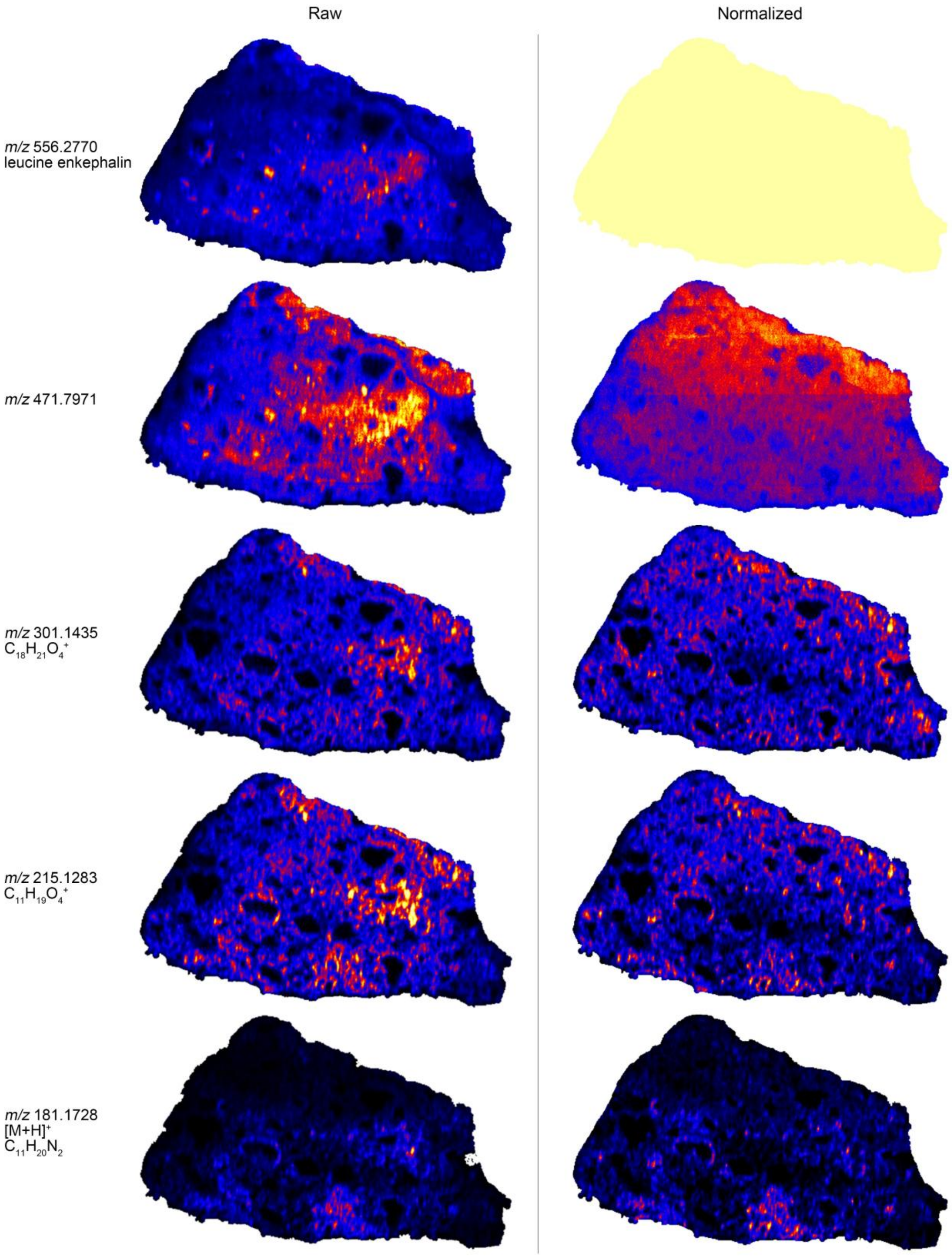

Figure S6. DESI-MSI of ions before and after normalization with lock mass (leucine enkephalin).

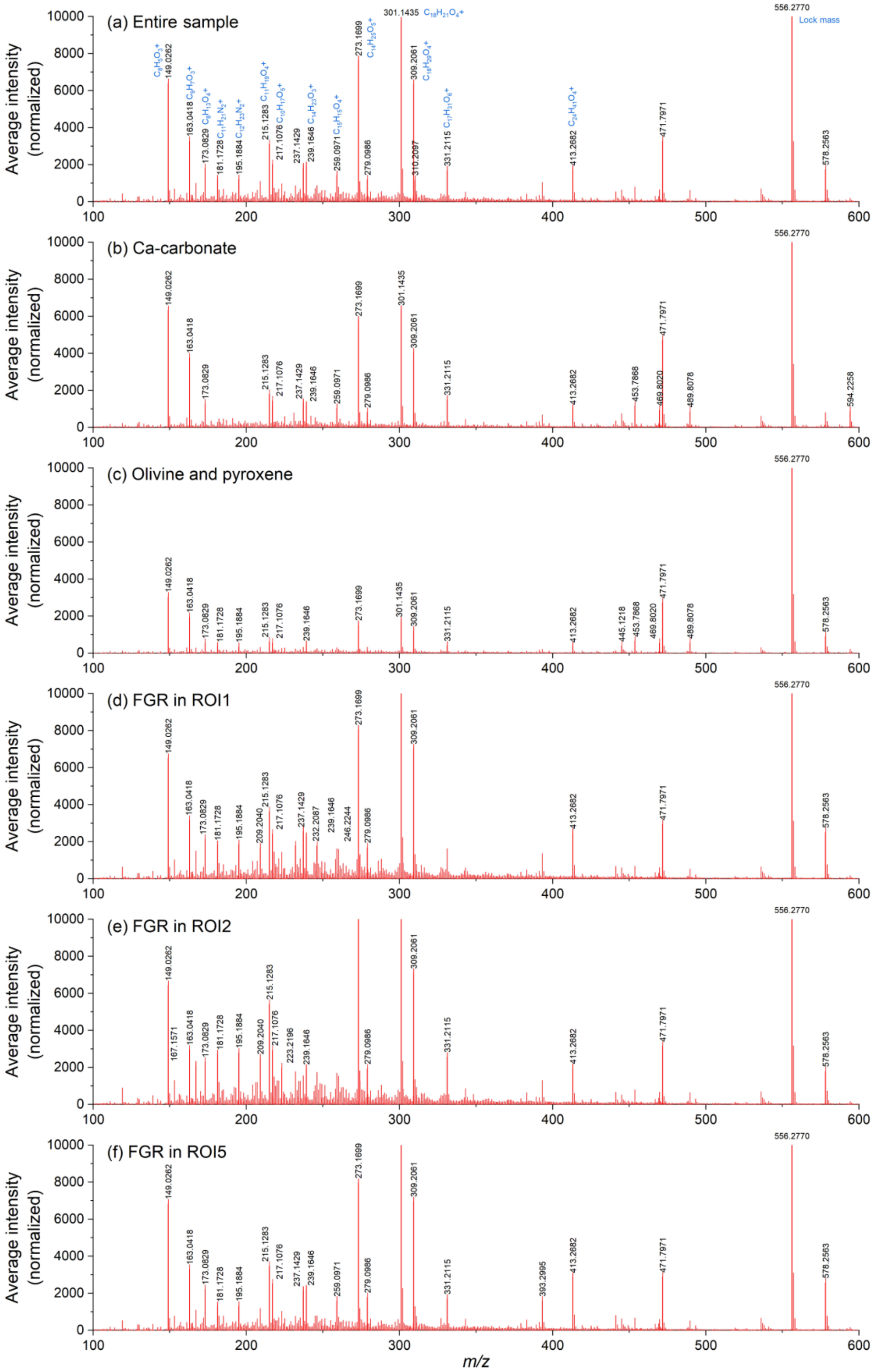

Figure S7. Average DESI-Q-TOF/MS spectra (normalized with lock mass) of the entire sample and different minerals.

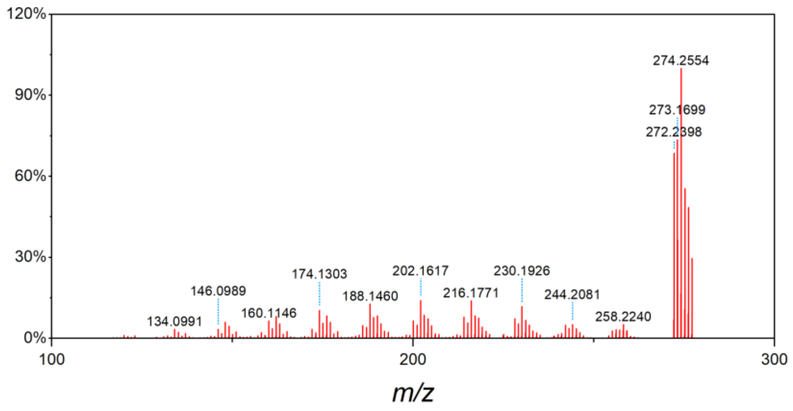

Figure S8. DESI-MS/MS spectrum of *m/z* 273.1699.

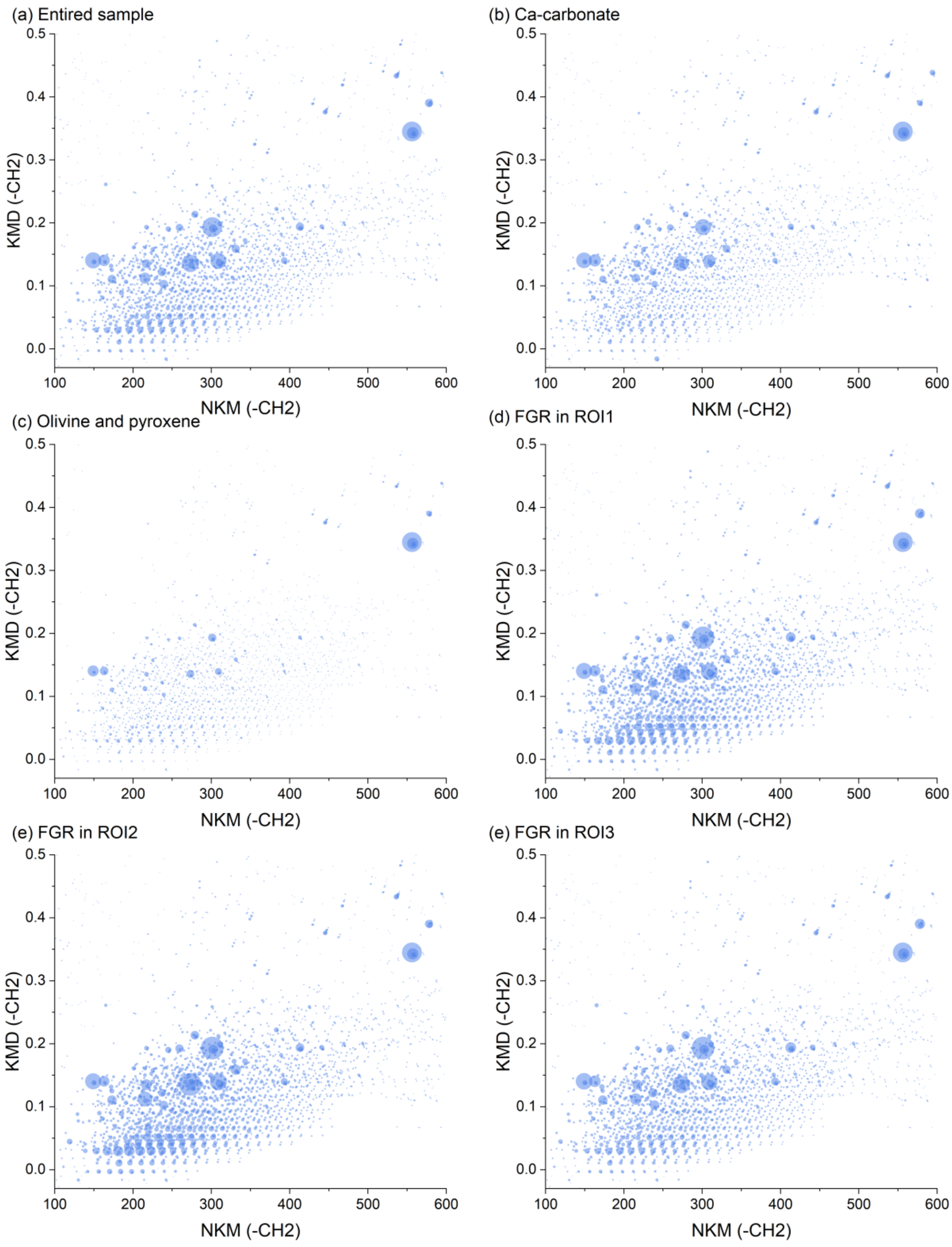

Figure S9. Kendrick mass defect (KMD) plot of DESI-Q-TOF/MS spectra. The circle area represents the relative abundance, and the scale ratio remains consistent in different panels. Horizontal axis (Nominal Kendrick Mass, NKM). Vertical axis (Kendrick Mass Defect, KMD).

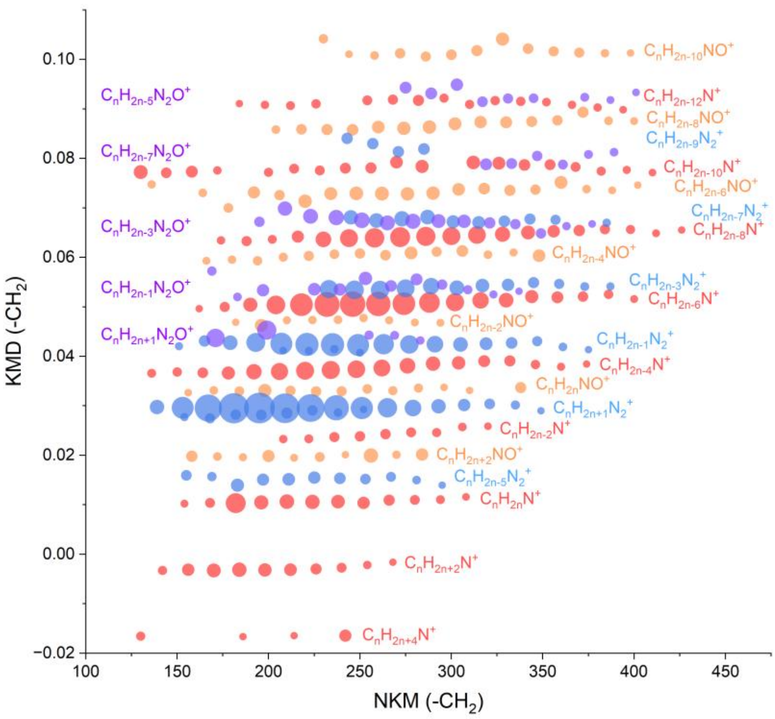

Figure S10. Kendrick mass defect (KMD) plot of DESI-Q-TOF/MS spectra and element composition assignments.

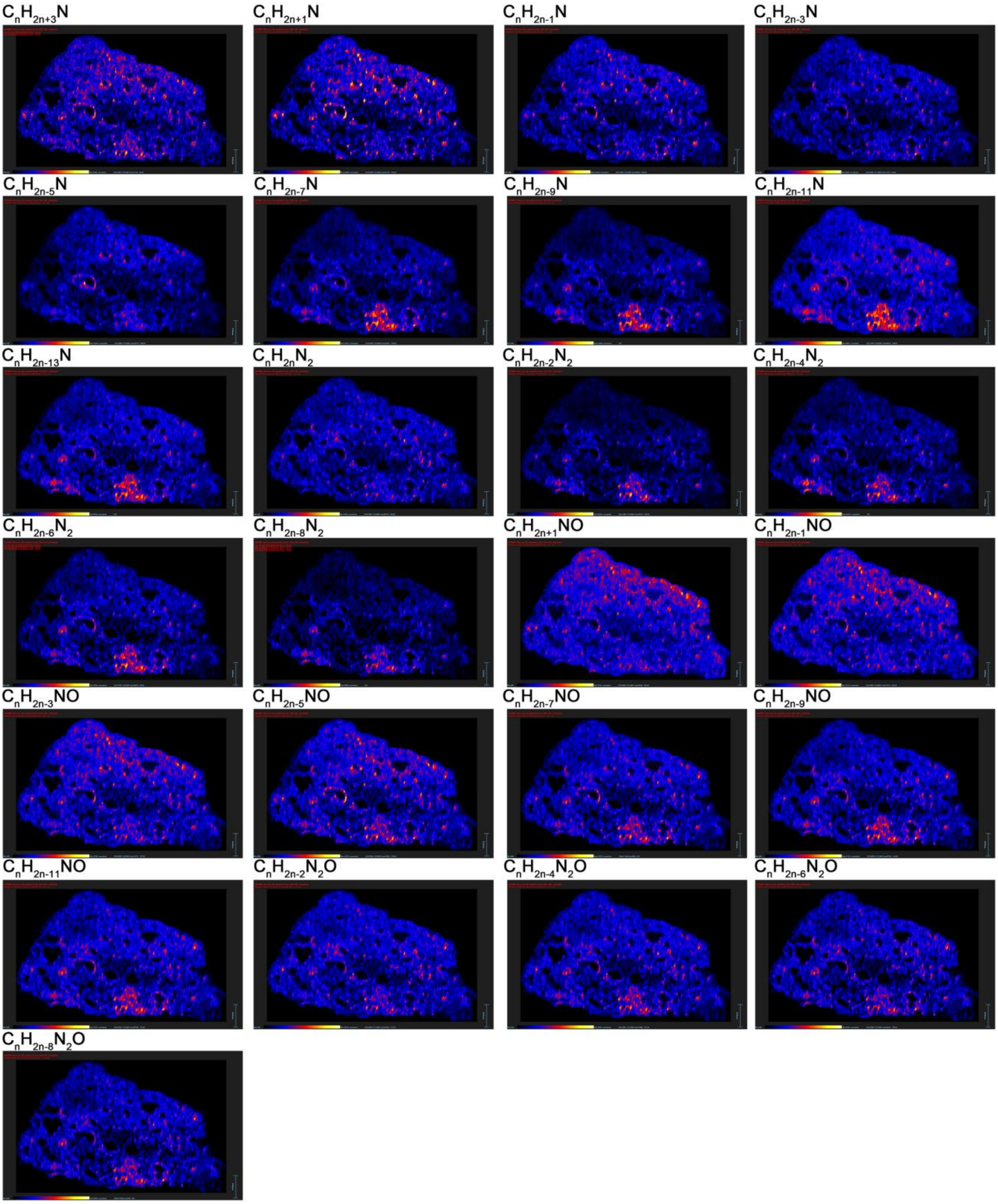

Figure S11. Spatial distribution of CHN and CHNO compounds. Intensity is given in the sum of alkylated homologues of CHN and CHNO species in Figure S11. The spatial distribution of each alkylated homologue of $C_nH_{2n-1}N$ and $C_nH_{2n-2}N_2$ is given in Figures S12 and S13.

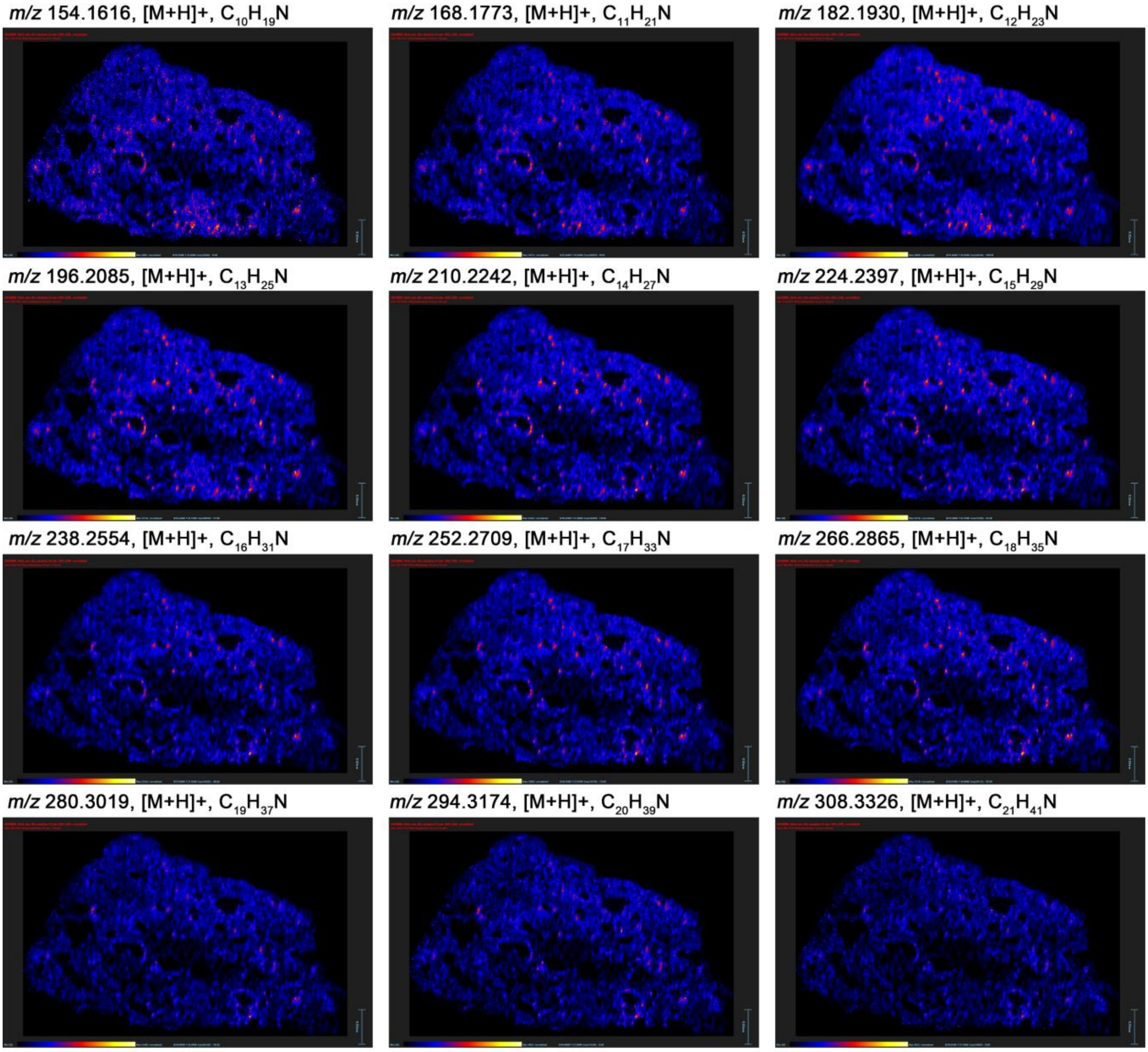

Figure S12. Spatial distribution of $C_nH_{2n-1}N$ alkylated homologues.

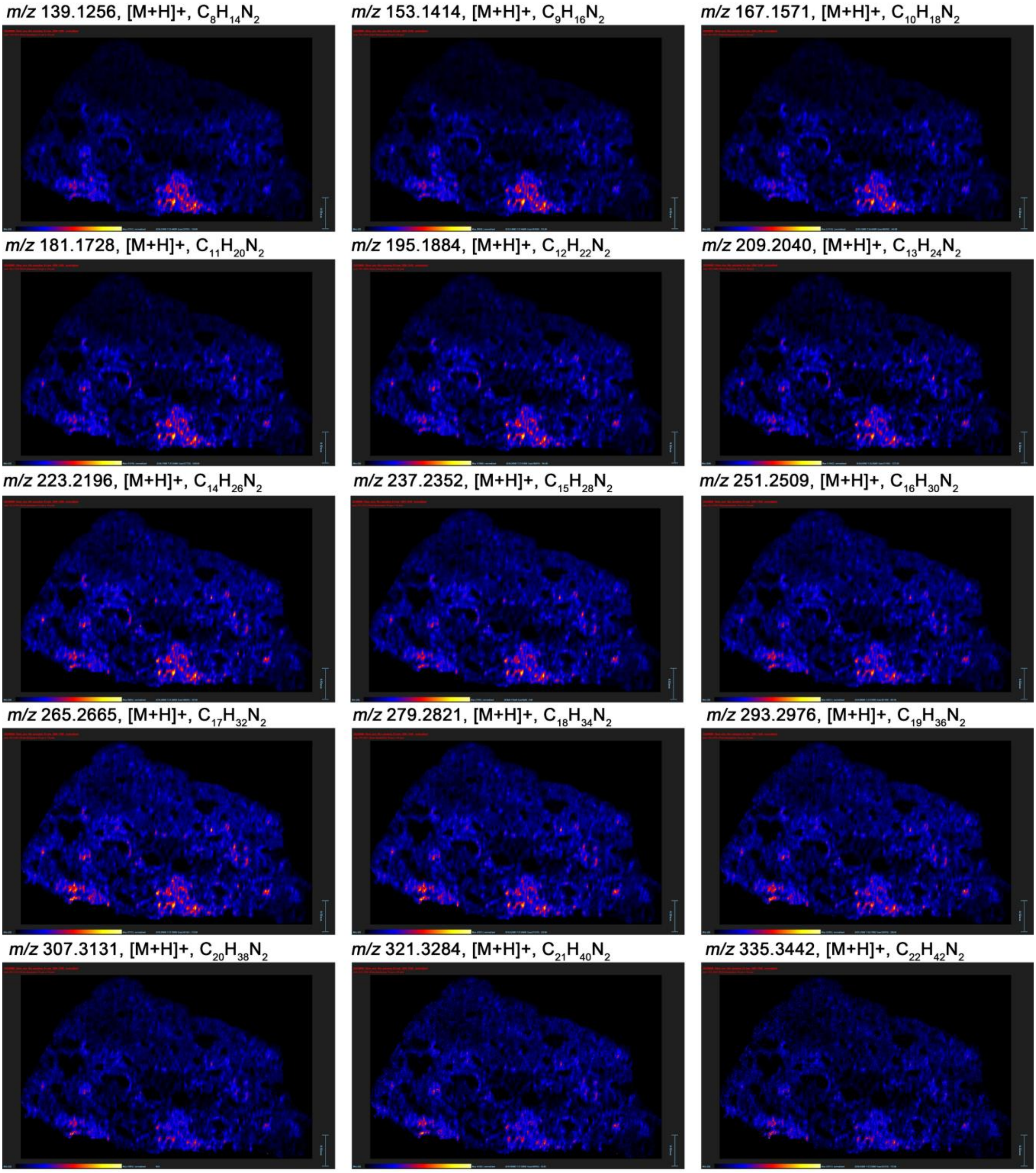

Figure S13. Spatial distribution of $C_nH_{2n-2}N_2$ alkylated homologues.

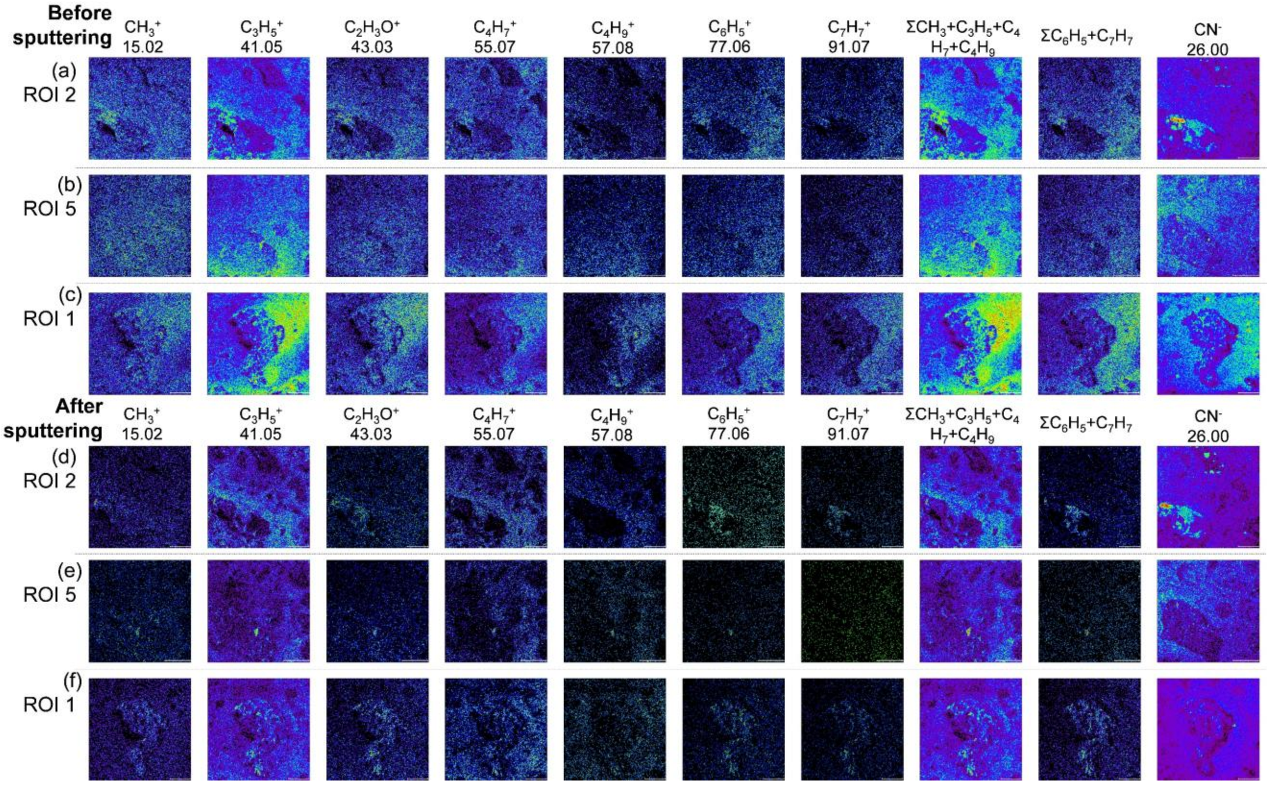

Figure S14. TOF-SIMS imaging of FGRs in the ROI before and after GCIB sputtering. All scales were 100 μm.

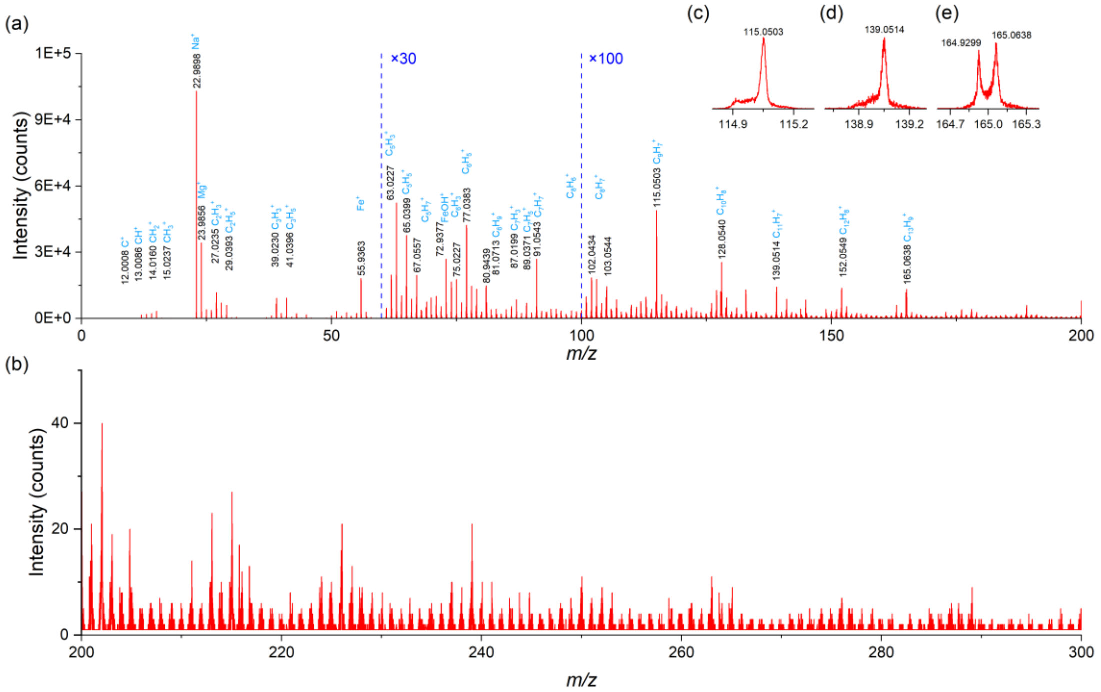

Figure S15. TOF-SIMS spectrum in positive mode.

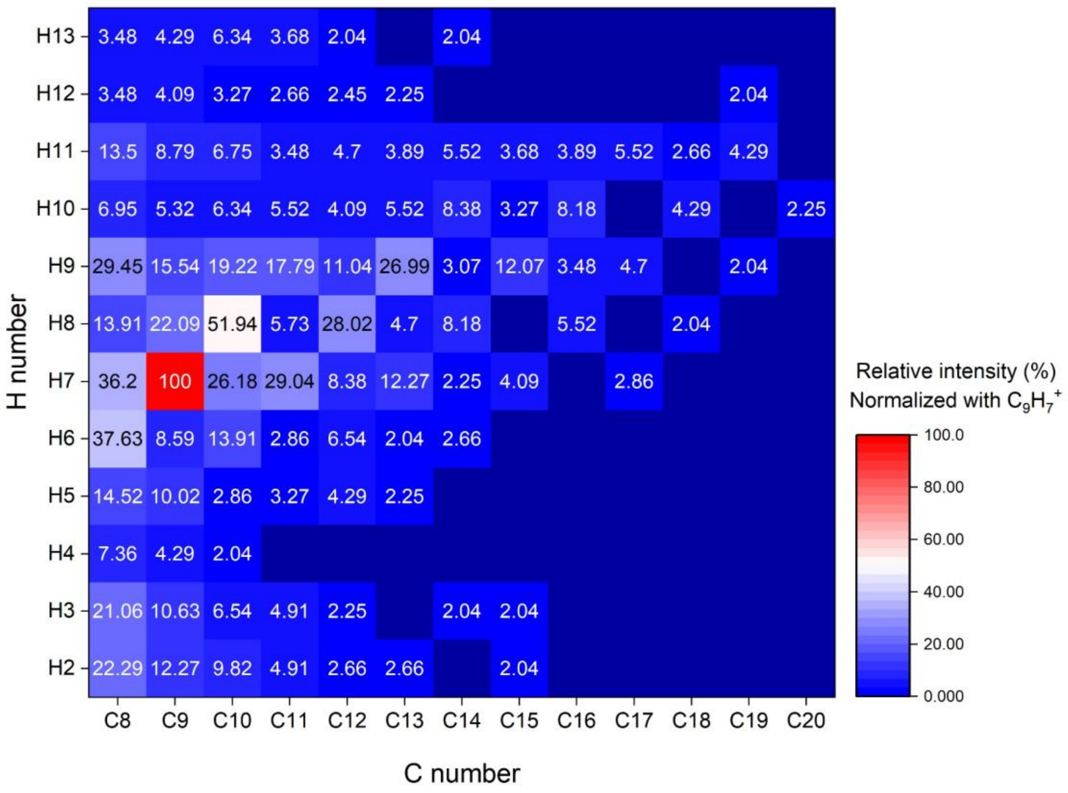

Figure S16. The C and H numbers of CH compounds in TOF-SIMS from m/z 98 to m/z 300 with a relative intensity (%) normalized with $C_9H_7^+$.

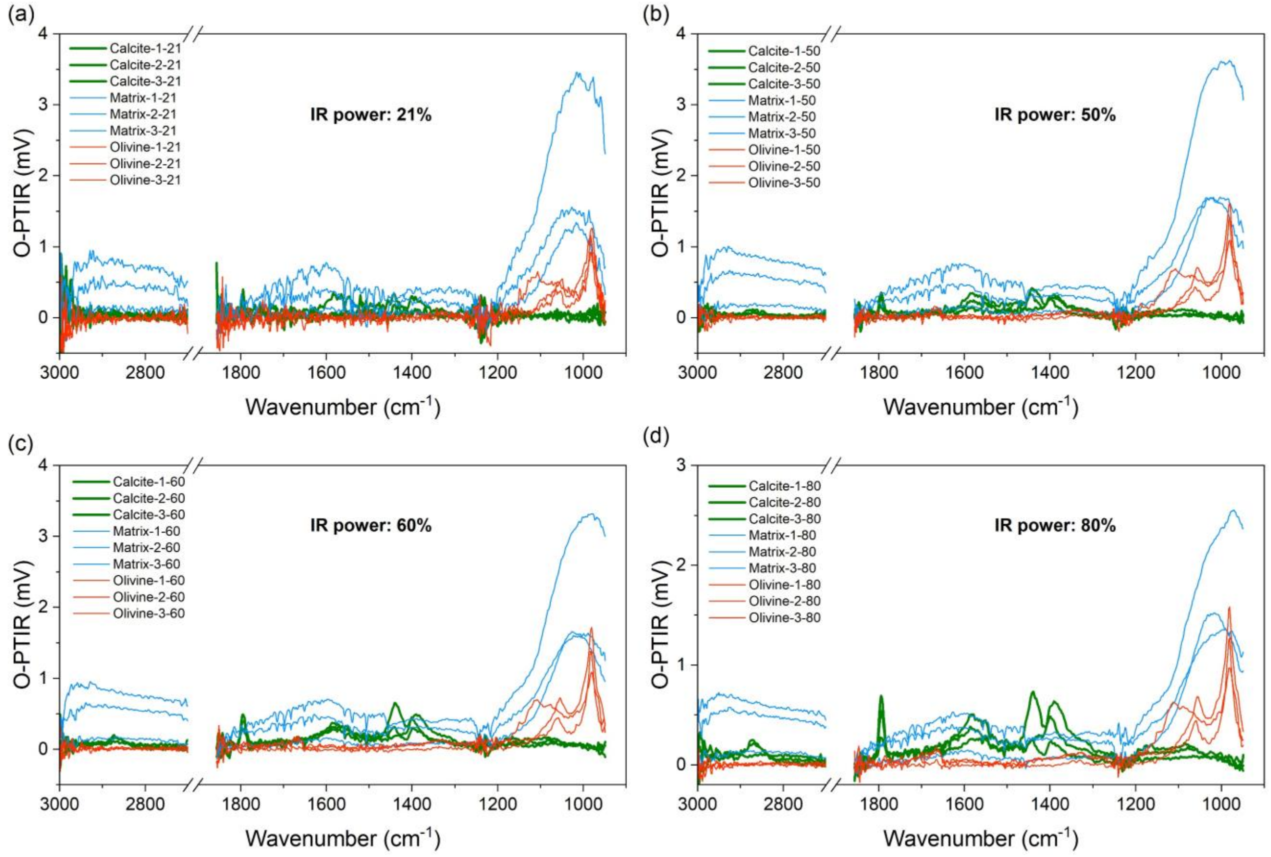

Figure S17. O-PTIR response for the different minerals under the same measurement conditions.

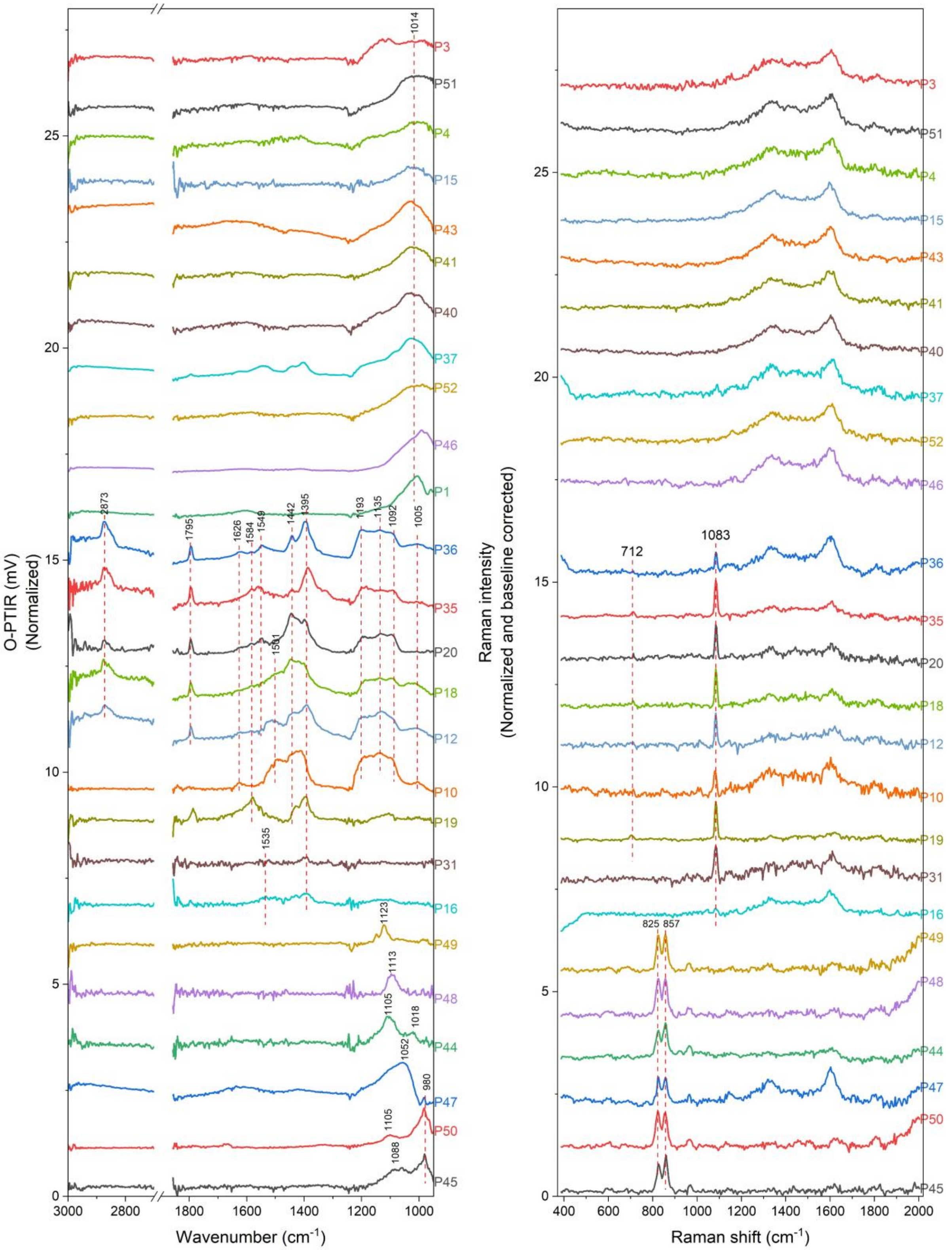

Figure S18. O-PTIR spectra and Raman spectra of calcite (P31, 19, 10, 12, 18, 20, 35, 36), olivine (P45, 50, 44, 48 and 49) and phyllosilicates (P1, 46, 52, 40, 41, 43, 15, 4, 51 and 3). P47 is close to the edge between the phyllosilicates and olivine. P37 is close to the edge between the calcite and matrix phyllosilicates.

References

(1) Hugenschmidt, M.; Adrion, K.; Marx, A.; Müller, E.; Gerthsen, D. Electron-Beam-Induced Carbon Contamination in STEM-in-SEM: Quantification and Mitigation. *Microscopy and Microanalysis* **2023**, *29* (1), 219-234. DOI: 10.1093/micmic/ozac003.

(2) Lin, Y.; El Goresy, A.; Hu, S.; Zhang, J.; Gillet, P.; Xu, Y.; Hao, J.; Miyahara, M.; Ouyang, Z.; Ohtani, E.; et al. NanoSIMS analysis of organic carbon from the Tissint Martian meteorite: Evidence for the past existence of subsurface organic‐bearing fluids on Mars. *Meteoritics & Planetary Science* **2014**, *49* (12), 2201-2218. DOI: 10.1111/maps.12389.

(3) Monico, L.; Rosi, F.; Miliani, C.; Daveri, A.; Brunetti, B. G. Non-invasive identification of metal-oxalate complexes on polychrome artwork surfaces by reflection mid-infrared spectroscopy. *Spectrochim Acta A Mol Biomol Spectrosc* **2013**, *116*, 270-280. DOI: 10.1016/j.saa.2013.06.084.

(4) Wang, Y.; Hsu, W. Petrology and mineralogy of the Ningqiang carbonaceous chondrite. *Meteoritics & Planetary Science* **2010**, *44* (5), 763-780. DOI: 10.1111/j.1945-5100.2009.tb00767.x.
